# Large-scale mapping of environmental-genetic interactions illustrates the dynamic nature of cell-cycle and DNA repair regulation

**DOI:** 10.1016/j.molcel.2026.01.025

**Published:** 2026-02-19

**Authors:** Benjamin W. Herken, Garrett T. Wong, Anna Mäkiniemi, Emma Lundberg, Thomas M. Norman, Luke A. Gilbert

**Affiliations:** 1 Tetrad Graduate Program, University of California, San Francisco, San Francisco 94518, USA; 2 Helen Diller Family Comprehensive Cancer Center, University of California, San Francisco, San Francisco 94518, USA; 3 Arc Institute, Palo Alto, CA 94305, USA; 4 Biological and Medical Informatics Graduate Program, University of California, San Francisco 94518, USA; 5 Science for Life Laboratory, School of Engineering Sciences in Chemistry, Biotechnology and Health, KTH - Royal Institute of Technology, Stockholm, Sweden; 6 Department of Bioengineering, Stanford University, Stanford, CA 94305, USA; 7 Memorial Sloan Kettering Cancer Center, New York 10065, USA; 8 Department of Urology, University of California, San Francisco 94518, USA; 9 Lead contact

## Abstract

Cells integrate exogenous and endogenous signals to grow, repair, or die. This is likely achieved through dynamic functional associations between genes, but measuring these relationships at scale is non-trivial. Here, we evaluate genetic associations in response to cell-cycle interruption, genotoxic perturbation, and nutrient deprivation using conditional genetic interaction (GI) mapping in human cells. In five maps measuring 250,000 GIs or higher-order environmental interactions, we discover widespread rewiring of relationships between genes, complexes, and ontologies across conditions. Specific bioprocesses drive the rewiring signal in each environmental state, as highlighted in our findings that the TIP60 and PP2A complexes radically alter their interaction profiles after inhibition of ATR. This resource reveals numerous genetic relationships for the fields of DNA damage signaling, DNA repair, and cell-cycle control and explores their context specificity. Our work advances a framework for using GI maps to explore environmental rewiring.

## In brief

To date, genetic interaction (GI) maps in the context of environmental perturbations have been limited to yeast studies. Herken et al. present five human GI maps that reveal how the landscape of genes’ functional relationships is rewired as cells experience environmental stress to DNA integrity, cell-cycle regulation, and metabolism.

## Graphical Abstract

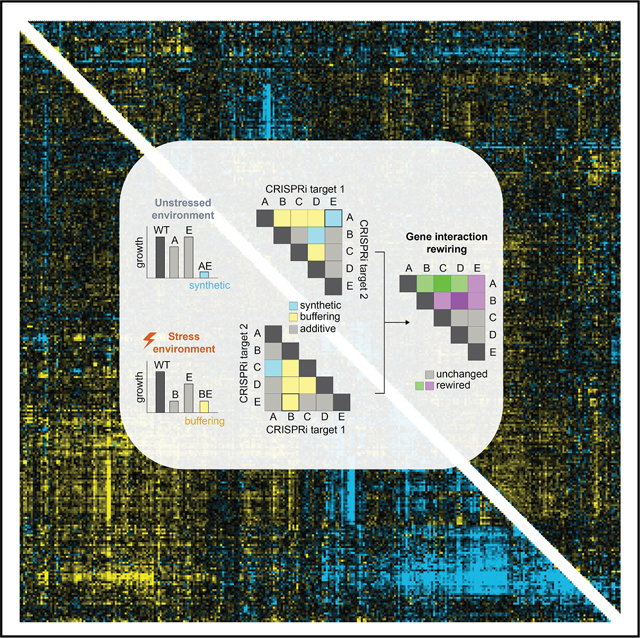

## INTRODUCTION

Drawing functional connections between individual genes, pathways, and processes is foundational to our understanding of the interdependence and redundancy of cellular functions.^1–8^ While most studies that seek to nominate putative associations between genes are limited to the basal environmental condition in which their model system is maintained, it is equally important to profile how these associations may change in different cellular states and contexts.

One such functional genomics approach that provides a scalable snapshot of gene relationships is measuring genetic interactions (GIs) between gene pairs. Gene pairs that genetically interact are usually found to be related or are components of distinct biological processes whose cooperation is (sometimes unexpectedly) important for cellular homeostasis.^9,10^ GI occurs when a combination of genetic perturbations elicits a phenotype that quantitatively differs from an expectation based on the single gene perturbation effects. Classically, GIs are observed in cell viability experiments, where surprisingly deleterious or mitigated growth defects are classified as either synthetic sick/synthetic lethal (SS/SL) or buffering/epistatic interactions, respectively.^11–13^

GI mapping, in which a large matrix of GIs is systematically and quantitatively measured between sets of genes, enables high-throughput identification of human GIs. In a GI map, each gene’s interaction profile can be used as a multi-dimensional signature to form hierarchical clusters. Clustered genes are often found to exist in protein complexes, have similar roles, or belong to similar ontologies.^14,15^ GI mapping has been used successfully in various organisms to assign putative function to uncharacterized genes and discover pathways and protein complexes.^16^ However, only recently has GI mapping been extended to human cells through the advent of robust functional genomics approaches and technologies such as CRISPR.^17–27^

One persistent shortcoming in GI design is the assumption that gene functionality is more or less static. The historical difficulty of molecular genetics has necessitated such reductive reasoning, but this could also underlie contradictions in the literature explainable by context-specific phenotypes.^28,29^ Two landmark studies have systematically measured environmentally triggered genetic rewiring in yeast, where rewiring phenotypes were found to be enriched among genes implicated in the response to the specific environmental stressor; however, an examination of the nature of such tripartite interactions in human cells remains elusive.^30–33^

**Figure 1.**
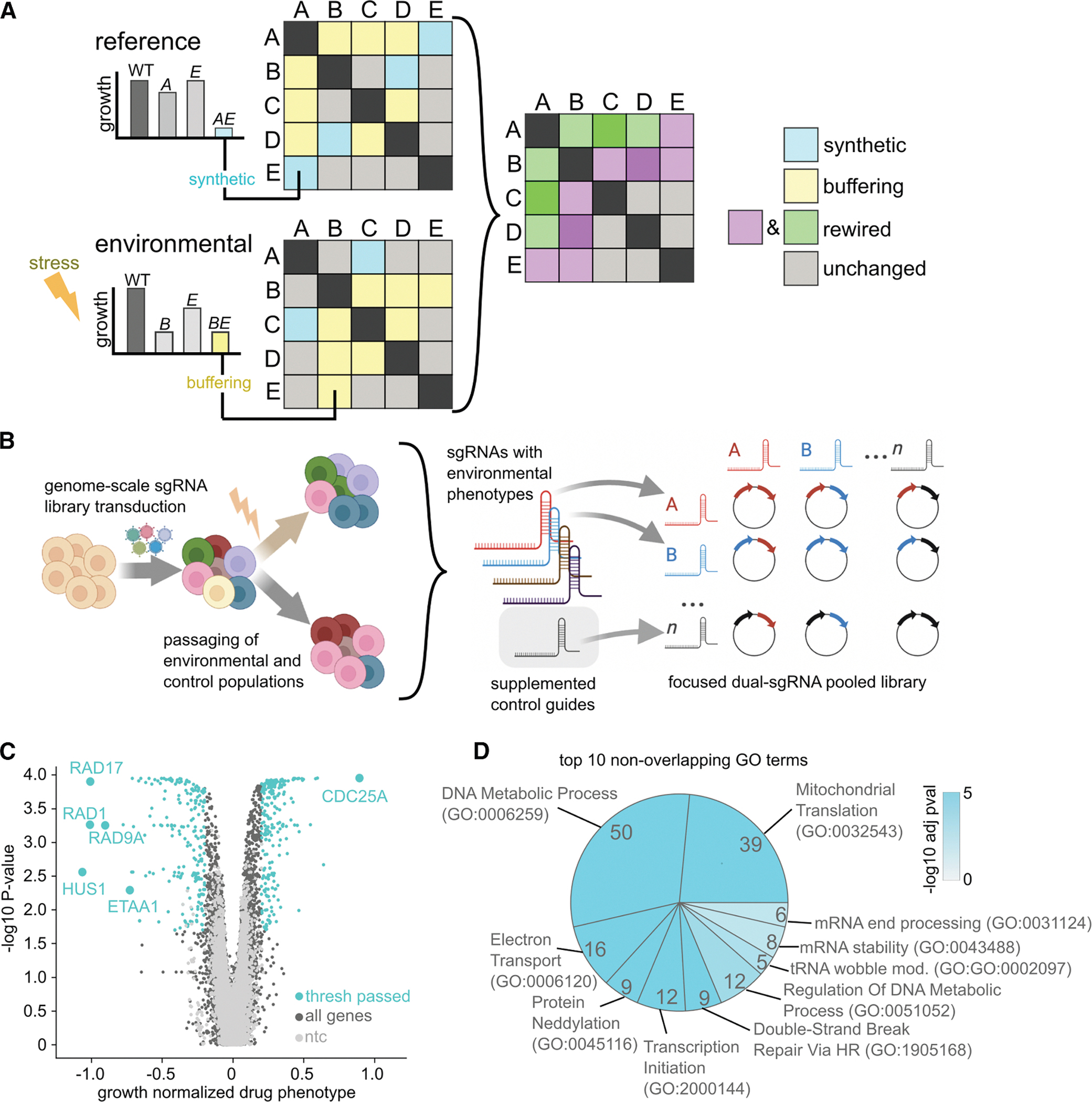
A high-fidelity CRISPRi screen for ATRi modulators (A) Schematic of dGI mapping to identify rewiring of gene or ontology relationships under environmental stress. Left: pairwise GIs under reference vs. environmental conditions. Middle: reference and environmental maps; blue, synthetic sick; yellow, buffering. Right: differential map highlights rewired interactions; green/purple, gain/loss of interaction. (B) Experimental workflow for genome-scale nominating CRISPRi screens in the presence/absence of perturbation to identify condition-specific hits. Validated sgRNAs are used to build dual-sgRNA libraries for GI mapping. (C) Volcano plot of gene-level growth normalized drug phenotypes and associated *p* values (Mann-Whitney) from the ATRi nominating screen in K562 cells. Significant hits (blue) meet statistical and phenotype thresholds. (D) GO term enrichment of ATRi screen hits. Terms shown are mutually exclusive and non-overlapping. Wedge width, hit count; color intensity, -log_10_ *p* value (KS).

Using CRISPR interference (CRISPRi)-based GI mapping in human cells, we investigate genetic rewiring across distinct environmental contexts: S-phase checkpoint inactivation, genotoxic stress from double-strand breaks, and glucose deprivation. We generate three environmental and two reference GI maps, each revealing robust, reproducible, and often unexpected phenotypes—including gene-by-gene (GxG) and GxG-by-environment (GxGxE) interactions. Each GI map provides a coherent snapshot of genetic architecture in a particular cell state, and comparison between maps identifies rewired GIs (Figure 1A). We find the composition and intra-cluster interaction profile of established ontologies is broadly conserved across conditions; however, the interactions between these ontologies are highly context-dependent. This dataset totaling 250,000 unique measured GIs provides a rich resource on cell-cycle regulation, DNA replication fidelity, DNA repair, and metabolism, highlighting unexpected biology relating to ATR, DNA topoisomerase, TIP60, and PP2A biology. Our work also establishes a framework for measuring and interpreting context-specific rewiring of human GIs.

## RESULTS

### A nominating CRISPRi screen identifies genes that modify sensitivity to ATR inhibition

We first aimed to identify genes that modulate sensitivity to perturbation of the ATR kinase, a key S-phase DNA damage checkpoint regulator. We reasoned that the DNA damage response (DDR), being environmentally reactive, was a fitting context for an examination of human GI rewiring. We conducted a genome-scale CRISPRi screen in K562 human leukemia cells ±750 nM ATR inhibitor AZD6738 (ATRi), using our established pipeline (Figure 1B; Table S1).^34,35^ This ATRi dose impaired growth but was not uniformly lethal. Replicate screens showed highly reproducible sgRNA and gene-level phenotypes in both control and ATRi-treated conditions (Figures S1A and S1B).

Many key genes essential for ATR function or known ATRi modulators were recovered in our hit list,^36–38^ including all three 9–1-1 complex members (RAD9A, HUS1, and RAD1) and its loader RAD17—as the top four genes whose knockdown increases ATRi sensitivity (Figure 1C). CDC25A, whose loss protects against ATRi, emerged as the strongest resistance hit.^39^ The presence of these positive controls, along with strong enrichment for DNA repair, cell-cycle, and chromatin-related Gene Ontology (GO) terms, supports the quality of this gene set as a starting point for mapping GIs related to cell-cycle and DNA repair biology (Figure 1D). Beyond expected DNA repair and regulatory factors, hits also include genes with GO terms unrelated to canonical ATR biology—such as glycolysis, neddylation, translation initiation, and mRNA stability (Figure 1D; Table S1)—suggesting the potential for a GI map to uncover unpredictable interactions among disparate cellular processes. From this nominating screen, we selected 293 genes conferring selective sensitivity or resistance to ATRi for inclusion in our GI mapping sgRNA library.

### Reference GI and ATRi eGI maps of S-phase checkpoint biology

We selected the top two sgRNAs per hit gene from the nominating screen to build a pooled dual-sgRNA GI library (Figure 1B; Table S2), including all sgRNA combinations, as described previously.^24^ The final library contains 408,321 sgRNA pairs covering 46,208 unique dual-gene perturbations, with eight constructs per gene pair (Figure S1C).

We conducted parallel CRISPRi GI screens in K562 cells using this library (Figure S1D), one under basal conditions (DMSO) and one dosed with 1.25 μM ATRi. Each condition included two biological replicates (Figures S1E and S1F).

Each gene perturbation is measured 52 times as each sgRNA is paired with a panel of non-targeting controls (ntc), enabling robust single-gene phenotype estimation—essential for accurate GI detection. Single-gene effects from both GI screens correlate strongly with those from the nominating screen (Figures S2A and S2B).

**Figure 2.**
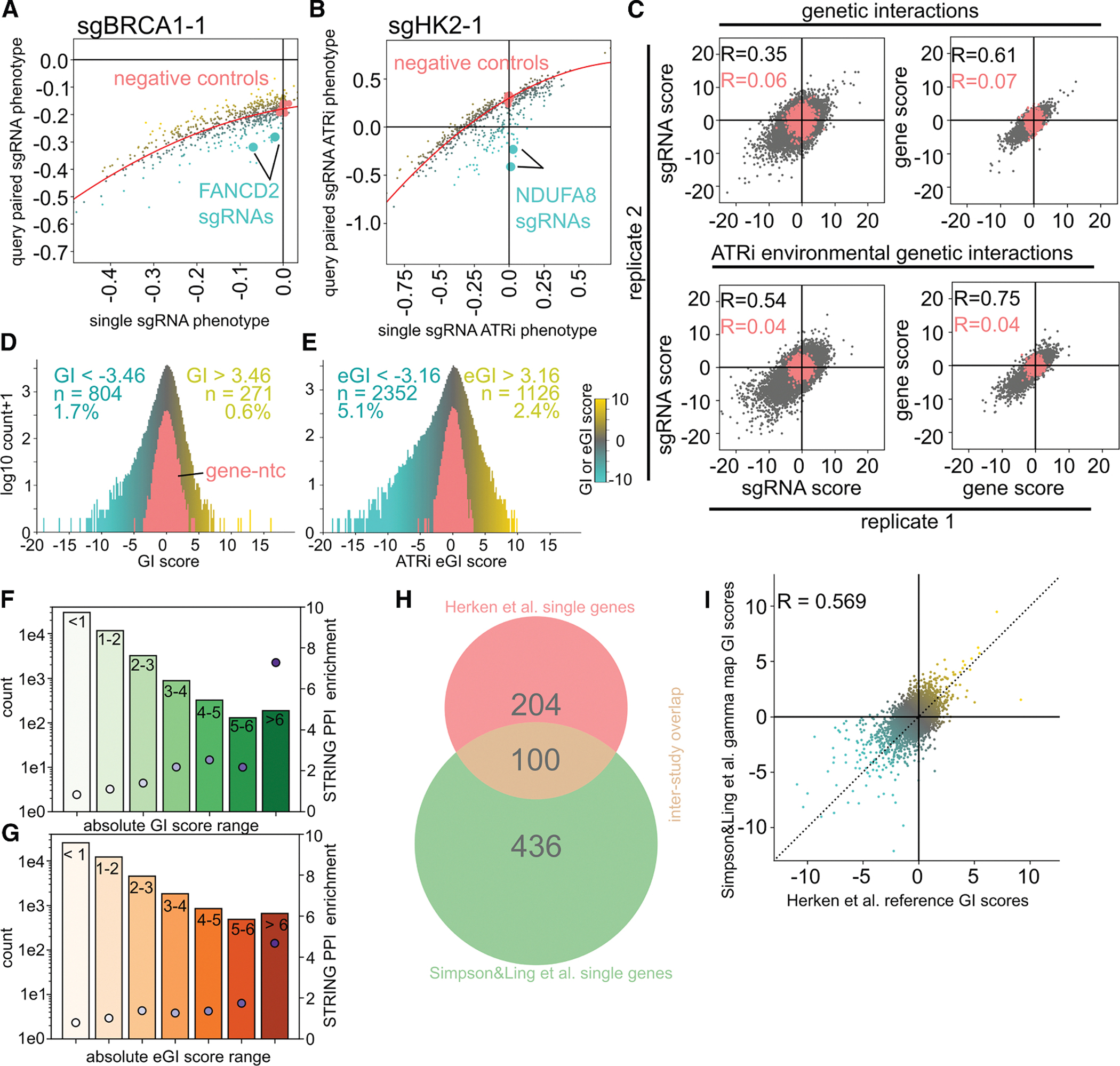
Calling GIs under variable conditions (A and B) Modeling and GI calling for example query sgRNAs: BRCA1 in reference (A), HK2 under ATRi (B). Pink, negative control distribution for the query sgRNA; red, regression model from single vs. query-paired sgRNAs. Highlighted: two sgRNAs for a known interactor. (C) Correlation of sgRNA- and gene-level GI scores between replicates in reference and ATRi conditions. Pink, ntc distributions. (D and E) Distribution of GI scores for all gene pairs in reference (D) and ATRi (E) maps. Blue, synthetic sick; yellow, buffering; pink, gene-ntc controls. GI and eGI high confidence thresholds set at four standard deviations of the negative control distribution. (F and G) STRING PPI enrichment by binned absolute GI (F) or eGI (G) scores. Dot plot, enrichment; bar, bin size. Chi-squared *p* values: reference = 1.98e–27; ATRi = 1.99e–19. (H) Venn diagram showing overlap of GI library genes from this study vs. Simpson and Ling. (I) Scatterplot of overlapping gene pair scores from our reference map vs. Simpson and Ling’s “gamma” map.

We generated a matrix of sgRNA-level GIs using a previously established method for mammalian GI mapping.^24^ For each sgRNA, a regression model derived from dual and single-sgRNA effects is used to calculate GIs, based on deviations from expected pair phenotypes (Figures 2A and 2B). GI scores are normalized by the sgRNA’s ntc distribution.

Gene-level GIs are then computed by averaging all sgRNA-level interactions for each gene pair. Since ATRi-treated interactions reflect GxGxE, we term them environmental-GIs (eGIs), in contrast to the DMSO-derived reference GIs (Table S3 and S4). The GI and eGI data are available to explore at https://parpi.princeton.edu/map/.

### Evaluating quality and reproducibility of reference GI and ATRi eGI datasets

We assessed the validity and reproducibility of our GI data using an established framework.^24,40^ First, we observed a strong correlation between biological replicates for both reference and ATRi eGI maps at the sgRNA and gene levels (Figure 2C). Second, to evaluate signal-to-noise, we defined significant interactions as those exceeding four standard deviations from the negative control GI distribution, which yielded 271 (0.6%) positive and 804 (1.7%) negative high-confidence GIs in the reference condition and 1,126 (2.4%) positive and 2,352 (5.1%) negative eGIs under ATRi treatment (Figures 2D and 2E). The rarity and density of GIs align with prior mammalian GI maps, supporting their biological validity.^24,40^ Third, we found no correlation between a gene’s primary growth or drug-response phenotype and the sign or strength of its GI or eGI score (Figures S2C and S2D), further supporting the robustness of our approach.

Fourth, since GIs often occur between physically interacting gene products, we examined overlap with known protein-protein interactions (PPIs) using the STRING database. Gene pairs with strong GIs were more likely to have predicted PPIs, especially in the reference condition, and this effect was weaker in the ATRi eGI map, likely because PPI databases reflect baseline conditions (Figures 2F and 2G). Many GIs and eGIs lacked PPI evidence, consistent with prior findings that GIs can arise from indirect or uncharacterized relationships.^24^

Fifth, an independent study using the same GI mapping method measured 4,950 overlapping gene pairs under reference conditions^41^ (Figure 2H). GI scores between studies were highly correlated (Figure 2I, R = 0.569), underscoring the fidelity of our dataset and the modularity of our approach.

**Figure 3.**
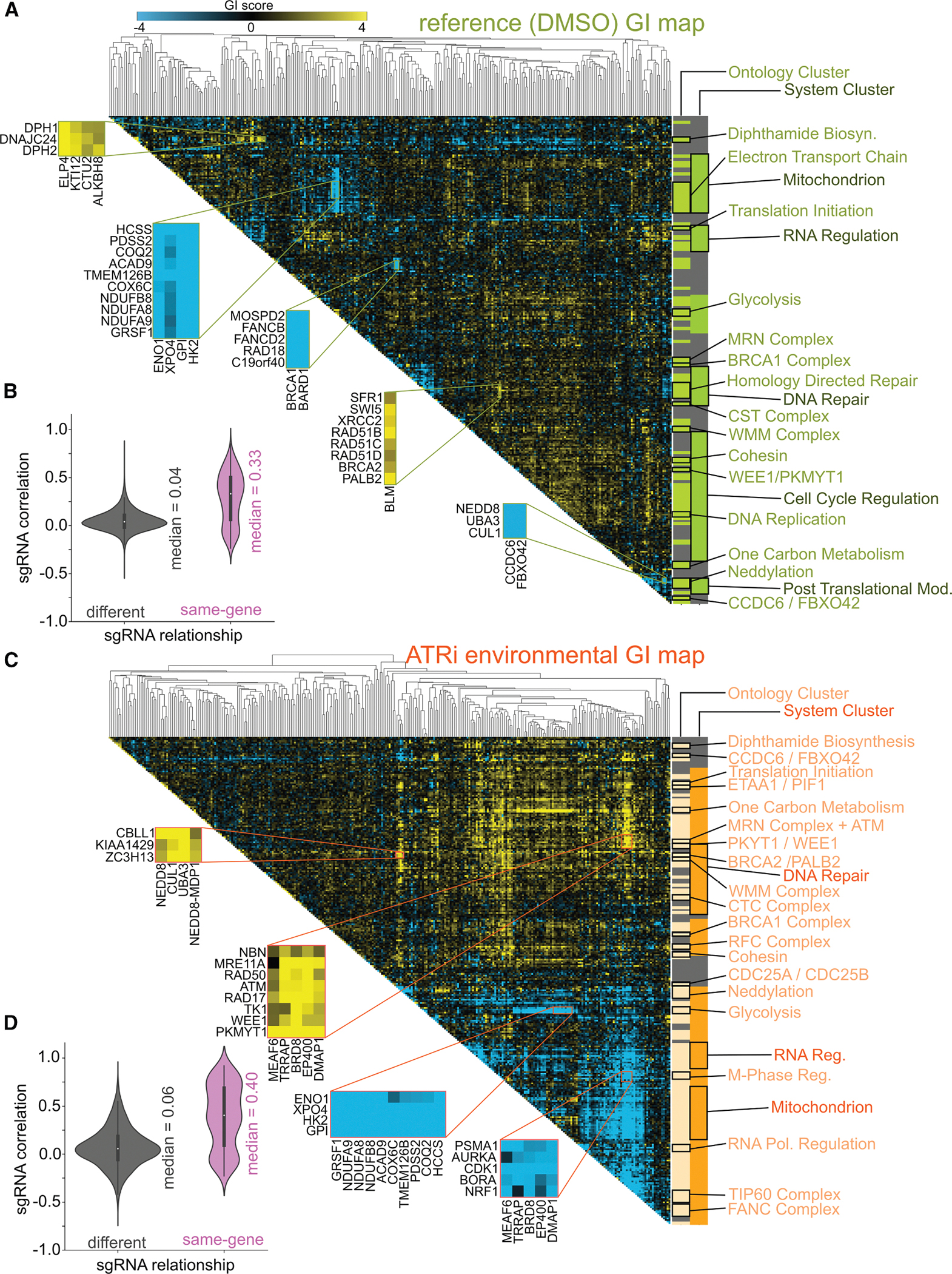
GI and eGI maps of cell cycle and DNA repair regulation (A and C) Heatmaps of gene-level GI scores under reference (A) and ATRi (C) conditions. Blue, synthetic sick; yellow, buffering. Dendrograms reflect average linkage hierarchical clusters of Pearson correlations. Light bars, high-confidence cluster (Pearson distance ≤ 0.55); dark bars, broader groupings (Pearson distance ≤ 0.80). (B and D) Violin plots of sgRNA-level Pearson correlation distributions for same-gene (purple) vs. different-gene (gray) pairs in reference (B) and ATRi (D) datasets.

Sixth, clustering genes by GI profile similarity provides a scalable, quantitative measure of functional relationships, as genes with similar profiles often share biological roles or complex membership. This allows use of known protein complexes and pathways as benchmarks for data quality. For each map, we applied hierarchical clustering using average pairwise Pearson correlation (Figures 3A and 3C), revealing robust groupings of functionally related genes. Small clusters (e.g., BRCA2/PALB2 and BRCA1/BARD1) reflect direct partners, mid-sized clusters (5–12 genes) capture known complexes like TIP60 and ETC complex I, and large clusters (∼100 genes) associate with compartments or processes (e.g., mitochondria and homologous recombination). To assess clustering fidelity, we compared correlations between sgRNAs targeting the same gene vs. unrelated genes. In both maps, same-gene sgRNAs were well correlated (Pearson R = 0.33 and 0.40 for GI and eGI), while unrelated ones were not (R = 0.04 and 0.06, respectively; Figures 3B and 3D).

Seventh, to further validate our analytic framework, a subset of GIs and eGIs were confirmed via fluorescence competition assays. In these assays, perturbed and wild-type (WT) cells are co-cultured, and fitness is assessed by tracking the depletion or enrichment of fluorescently labeled perturbed cells over time (Figures S3A–S3D). Additionally, some GIs and eGIs identified in K562 cells were conserved in A549 lung cancer cells, suggesting that, at least in the context of DNA replication and S-phase checkpoint biology, cell-line-specific interactions are relatively rare (Figures S3E–S3H).

### Using GI maps to generate biological hypotheses

The predictable clustering of genes in a GI map is an important internal benchmark but is particularly compelling when used to reveal associations involving poorly characterized genes. These groupings allow for rapid hypothesis generation based on shared interaction profiles. One striking example involves the karyopherin XPO4, which clusters closely with glycolytic enzymes (GPI, HK2, and ENO1). All four genes share strong negative interactions with mitochondrial oxidative phosphorylation factors in both the reference and ATRi eGI maps (Figures 3A and 3C). These findings were validated by fluorescence competition assays (Figures S3C and S3D), suggesting either a direct role for nuclear-cytoplasmic transport in glucose metabolism or a previously unrecognized metabolic function for XPO4.

Another illustrative cluster includes just two genes: CCDC6 and FBXO42. CCDC6, best known for its oncogenic fusion events, has also been implicated in DNA damage signaling via the phosphorylated histone variant γH2AX, though its mechanism remains unclear.^42^ FBXO42 is a member of the F-box protein family, which confers target specificity to SCF ubiquitin ligase complexes. This pair negatively interacts with core SCF components—including CUL1, UBA3, and NEDD8—in both GI and eGI maps (Figure 3A) and also exhibits strong synthetic interactions with PPP2R1A (Table S3), implicating a potential role in cell-cycle regulation through PP2A. Supporting this, knockdown of either gene in ATRi-treated K562 cells leads to depletion of cells in G1 phase, as measured by propidium iodide (PI) staining—an effect not seen in untreated controls (Figures S2E–S2G).

Taken together, these data support a model in which CCDC6 and FBXO42 act as regulators of G1 progression, likely via SCF-dependent degradation pathways. Loss of their function may promote unscheduled cell-cycle entry, which, in combination with ATR inhibition, results in catastrophic mitotic progression.

These examples—and the thousands of other GI and eGIs mapped here—can be explored interactively at https://parpi.princeton.edu/map/, providing a resource for generating targeted functional hypotheses.

### Differential interactions reveal rewiring of inter-complex functional connections

**Figure 4.**
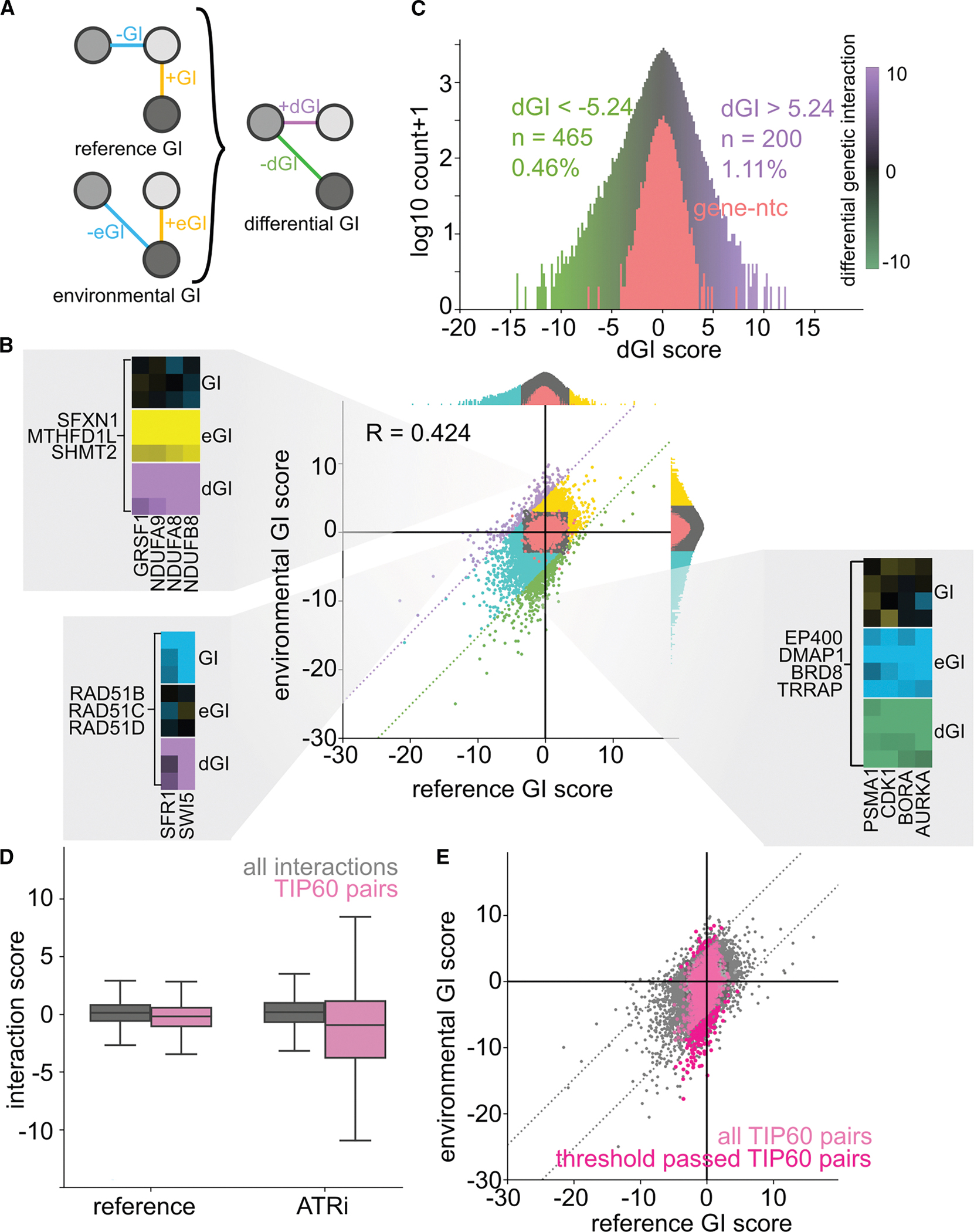
Analysis of dGIs induced by ATR inhibition (A) Schematic for deriving differential GIs (dGIs) by subtracting reference GIs from eGIs. (B) Scatterplot of gene-level GIs (*x* axis) vs. eGIs (*y* axis). Dotted lines indicate significance thresholds for dGI scores. Cutouts are used to highlight select conditional interactions: positive and negative dGIs are in purple and green, respectively; positive and negative GIs are in yellow and blue, respectively; and gene-ntc pairs are in red. (C) Distribution of ATRi dGI scores: positive and negtive dGIs are in purple and green, respectively; gene-ntc pairs are in red. (D) Boxplot of GI and eGI scores for TIP60 gene interactions (pink) vs. all interactions (gray); error bars denote standard deviation. (E) Scatterplot from (B) with TIP60 gene interactions in pink; high-confidence dGIs are in dark pink; dotted lines indicate high-confidence thresholds for dGI scores.

Inspection of our data revealed substantial divergence between the reference and ATRi GI maps, a finding that initially surprised us (Figures 3A and 3C). To determine whether these eGIs arose from amplification of existing phenotypes or reflected newly emergent interactions, we compared GI scores across conditions (Figure 4A). While the GI and eGI scores are often correlated (Pearson R = 0.42), many gene pairs exhibit significant interactions in only one condition. Notably, numerous interactions in the ATRi eGI map are undetectable in the reference GI map—even after accounting for differences in signal strength between conditions (Figure 4B). These findings prompted us to quantify differential GIs (dGIs), which could represent context-specific rewiring of genetic networks—a phenomenon that, to our knowledge, has not yet been systematically mapped in human cells. To this end, we constructed a differential interaction matrix by subtracting the GI score in the reference map from that in the ATRi eGI map for each gene pair (Table S5). This matrix removes interactions unaffected by ATRi and isolates those whose genetic relationships are specifically modulated by ATR inhibition (Figure 4A). We defined high-confidence dGIs as those exceeding five standard deviations from the distribution of gene-ntc dGI scores. Additionally, we excluded dGIs where neither of the original GI/eGI scores reached significance. Using these criteria, we identified 200 positive and 465 negative high-confidence dGIs (Figure 4C).

Differential interaction analysis offers a powerful lens to uncover context-dependent relationships between genes and ontologies—interactions that might otherwise escape attention. For example, in the reference GI map, RAD51 paralogs (RAD51B, RAD51C, and RAD51D) form a tightly connected cluster with other homologous recombination repair factors such as SFR1 and SWI5 (Figure 4B). This cluster exhibits strong negative intra-complex GIs, reflecting functional interdependence in managing replication stress. In the ATRi map, the cluster remains intact, but its broader connectivity to other DNA repair modules is disrupted. Many DNA repair sub-complexes lose their inter-cluster interactions and show fewer GIs with unrelated ontologies (Figures S4A and S4B). This erosion of network connectivity—sometimes referred to as “masking” of interactions^30^—is consistent with an ATRi-induced impairment in mobilizing homologous recombination, aligning with current models of the DDR.

Conversely, we also observe interactions that emerge only under ATR inhibition. For example, while a conserved metabolic cluster comprising MTHFD1L, SFXN1, and SHMT2—genes central to mitochondrial one-carbon metabolism—is not associated with the mitochondrial supercluster in the reference map, it shows strong positive eGIs under ATRi (Figure 4B). The conditional nature of this relationship, combined with the shared mitochondrial localization of the encoded proteins, suggests a model in which mitochondrial stress or energy depletion contributes to ATRi resistance.

Another striking trend is the prevalence of SL interactions in the ATRi map that are entirely absent in the reference condition. A significant number of these interactions involve components of the TIP60/NuA4 histone acetyltransferase (HAT) complex (Figures 4B, 4D, and 4E). TIP60 is a multifunctional chromatin modifier known to facilitate transcription, ATM activation, and histone exchange—particularly of the H2AZ and H2AX variants.^43–45^ Multiple TIP60 components are represented in our screen (DMAP1, EP400, BRD8, MRGBP, EPC2, and TRRAP). While all these genes cluster together in the reference GI map, only a conserved core of four—BRD8, DMAP1, EP400, and TRRAP—remains clustered in the ATRi map. This subtle restructuring is consistent with reports of TIP60 sub-complex specialization^46^ and may reflect a shift in functional dependencies under replication stress.

### Comparing eGI maps in similar and distinct environments illustrates coordinating ontologies that drive environmentally unique rewiring programs

We next sought to determine whether the extensive rewiring observed in the ATRi eGI map was a condition-specific phenotype or a more general response to cellular stress or toxicity. To address this and extend our analysis to distinct perturbations, we conducted three additional GI mapping experiments in K562 cells using the same dual-sgRNA CRISPRi library. These included a second reference DMSO GI map, an eGI map with the genotoxic chemotherapeutic etoposide, and an eGI map generated under glucose deprivation. Etoposide functions by inhibiting and trapping topoisomerase II on DNA, thereby blocking replication forks and inducing double-strand breaks.^47^ We hypothesized that the cellular response to etoposide would engage many of the same DNA repair and cell-cycle pathways as ATR inhibition, while also allowing us to distinguish shared from perturbation-specific rewiring events. By contrast, glucose deprivation represents a metabolic challenge distinct from DNA damage, providing an orthogonal environmental context that could reveal rewiring in energy-related pathways. All three new GI maps were generated as two independent biological replicates with similar reproducibility to the two previous conditions (Table S6, S7, and S8). Gene-level replicate correlations were robust across conditions (Pearson R = 0.63 for DMSO, 0.64 for etoposide, and 0.55 for glucose deprivation; Figures S4C–S4E). Applying the same high-confidence threshold (four standard deviations from the gene-ntc distribution), we identified 191 positive and 548 negative GIs in the DMSO map, 774 positive and 698 negative GIs in the etoposide map, and 187 positive and 434 negative GIs in the glucose deprivation map (Figures S5A–S5C).

**Figure 5.**
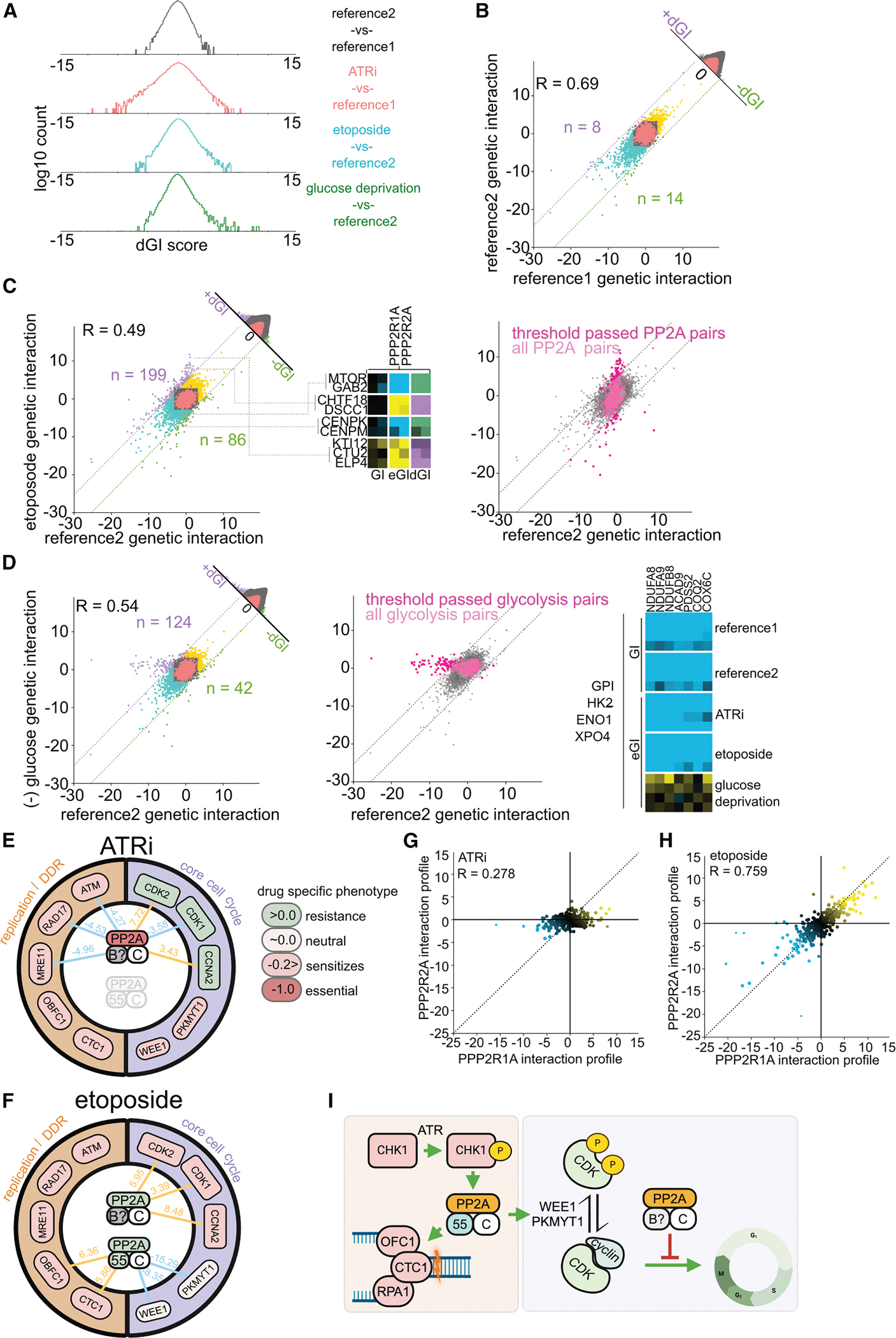
GI and eGI maps under reference, etoposide, and glucose deprivation conditions (A) Distribution of dGI scores for all conditions: reference (black), ATRi (red), etoposide (blue), and glucose deprivation (green). (B) Scatterplot of interactions measured between reference maps. The diagonal histogram shows corresponding dGI scores as in Figure 4B. Blue/yellow regions, synthetic sick/buffering interactions. Gene-ntc pairs in red. Dotted lines indicate high-confidence thresholds for dGI scores. (C) Left: eGI vs. reference GI scatterplot for etoposide as in Figure 4B. Middle: PP2A genes vs. select conditionally interacting genes. Right: all PP2A GIs in magenta; threshold-passed dGIs in a darker shade. (D) Left: eGI vs. reference GI scatterplot for glucose deprivation as in Figure 4B. Middle: glycolysis-related GIs highlighted in magenta; significant dGIs in a darker shade. Right: interaction patterns between glycolysis and oxidative phosphorylation genes across all conditions. (E and F) Conditional interaction networks and phenotypic patterns for PP2A and key interactors. Blue/yellow edges, buffering/synthetic sick interactions. For panels including B55 alpha (bottom), values are averaged across both PP2A complex members. The color key indicates the primary growth phenotype per condition. (G and H) eGI profile correlations of PPP2R1A vs. PPP2R2A under ATRi (G) and etoposide (H). (I) Model: conditional roles of PP2A subcomplexes. B55 alpha-associated PP2A is activated by CHK1 phosphorylation and stabilizes WEE1/PKMYT1 and CST. B55 alpha-independent PP2A counteracts CDK/cyclin complexes to suppress cell cycling.

GI scores from the second reference map were highly correlated with those from the first reference (Pearson R = 0.69; Figures 5A and 5B), providing further confidence in the stability of core GI profiles. Importantly, only 22 gene pairs exceeded the significance threshold for dGIs between the two reference maps, suggesting that high-confidence dGIs reflect true context-dependent functional differences rather than technical noise.

Comparison of GI and eGI scores between the second reference map and the etoposide and glucose deprivation maps showed concordance (Pearson R = 0.49 and 0.54, respectively; Figures 5C and 5D), but as in the ATRi condition, we observed substantial subsets of interactions unique to either the environmental or reference condition. We thus constructed two new dGI matrices representing the difference in interaction scores for the etoposide and glucose-deprived maps relative to the DMSO reference (Table S9 and S10).

The etoposide-specific dGI map revealed several ontologically consistent rewiring events. For example, members of the CHTF18-RFC complex—implicated in sister chromatid cohesion and replication fork protection^48,49^—interact negatively with BRCA1/BARD1 and CST complex factors also involved in replication fork stability and restart^50^ in our reference dataset. These interactions form a block that appears to be mediated by a shared responsibility in maintaining replication fidelity. Interestingly, these interactions are all lost in the etoposide condition, and, instead, the CST complex aligns closely with stress response mediators and core cell-cycle regulatory factors such as MAPKAP1 and the PP2A complex (Figure 5C; Table S9).

On further investigation, the most striking results from the etoposide-treated dataset are the pervasive positive eGIs formed between the PP2A complex and diverse functional modules, including DNA replication, fork stabilization, spindle assembly, and translation (Figure 5C). In each case, genetic perturbation of PP2A alleviated the otherwise deleterious effects of disrupting these ontologies, suggesting that PP2A coordinates a conditional network of dependencies under replication stress. This echoes the role of TIP60 observed in the ATRi condition, supporting a model in which central regulatory complexes shift their connectivity in response to environmental pressures.

The glucose deprivation eGI map correlated well with the DMSO reference map (Figure 5D), suggesting broad conservation of the GI signal. However, we observed a distinct and dramatic exception: a suite of strong negative GIs/eGIs present in all other maps—including both reference maps and the ATRi and etoposide conditions—was entirely absent in the glucose-deprived condition. These missing interactions almost exclusively involve gene pairs comprising glycolytic enzymes and genes involved in oxidative phosphorylation or mitochondrial homeostasis. We interpret this as a case of environmental epistasis: in the absence of glucose, glycolytic genes are effectively inactive, uncoupling their functional relationships with other metabolic processes. As a result, the glucose deprivation map produces a remarkably specific set of dGIs, with nearly all other interactions preserved from the reference map.

To validate our findings across all three environmental conditions, we constructed a focused ∼8,000-element dual-guide CRISPRi library targeting genes with minimal phenotypes in the primary screens. This “neutral” background allowed us to test a small number of gene pairs predicted to exhibit strong conditional interactions. Using this validation library in the reference and three environmental conditions, we generated single- and dual-gene phenotype matrices (growth normalized log2 fold enrichment scores, see STAR Methods) that correlated well with the original data (Pearson R = 0.917, 0.854, 0.681, and 0.794 for the reference, ATRi, etoposide, and glucose deprivation conditions, respectively; Figures S5D–S5G). We used these models to confirm specific interactions, including an etoposide-specific negative interaction between GAB2 and MTOR, an etoposide-specific positive interaction between CSRP2BP and BRCA2, and a negative interaction between WDHD1 and ETAA1 that was strongest in glucose deprivation but also present in the reference map (Figures S5H–S5J).

To control for potential batch effects, we also recalculated dGIs using unmatched reference maps (reference map 2 for ATRi; reference map 1 for etoposide and glucose deprivation). These analyses yielded highly similar results (Pearson R = 0.835, 0.731, and 0.656 for ATRi, etoposide, and glucose deprivation, respectively; Figure S5K), further supporting the validity of our approach.

### Differential interaction mapping guides mechanistic insight of complex biology

One goal of mapping changes in GI is to disambiguate functions of complicated, multifaceted protein complexes with annotated activities involving a range of sometimes contradictory functions. PP2A is one such complex that is broadly considered to function as a tumor suppressor and is often found in a repressed state in various cancers.^51^ By comparing the behavior of core and regulatory subunits of PP2A across conditions, we can start to use dGIs to separate distinct signaling control mechanisms and infer how they are wired under different types of stress. In the case of ATR inhibition, we observed the loss of genetic association between the scaffolding PPP2R1A and regulatory PPP2R2A (B55 alpha) subunits (Figure 5E). This points to a reorganization of the complex that likely reflects altered regulatory requirements or availability of subunit partners. The absence of strong B55 alpha-mediated interactions in this condition, especially with checkpoint and replication stress response genes, suggests that ATR activity may be required for B55 alpha incorporation or stability. Conversely, in etoposide-treated cells, where replication-associated DNA damage is pervasive and checkpoint activity is likely intact, PP2A and B55 alpha maintain coherent function, especially in support of fork protection and G2/M control (Figure 5F). Underscoring the severed functionality between the scaffold and regulatory subunits, the interaction profiles of PPP2R1A and PPP2R2A are poorly correlated in the ATRi conditions, where the profile of PPP2R2A is found to be significantly interrupted relative to PPP2R1A (Figure 5G). However, in the etoposide condition, the profile of these two genes is highly consistent with one another, suggesting a conserved functionality (Figure 5H).

The distinct interaction profiles of PP2A components across conditions also underscore how functional modules within the same complex can become differentially engaged, potentially through post-translational modification, differential expression, or competition among regulatory subunits. For example, the persistent interactions between PPP2R1A and CDK/cyclin genes suggest a basal PP2A activity that may be constitutively present and independent of regulatory subunit mediation. The observation that these interactions invert their phenotypic outcome depending on context (sensitizing in ATRi, protective in etoposide) highlights the nuanced interplay between phosphatase activity and cell-cycle state, particularly under genotoxic vs. checkpoint-disabling stress.

We propose that PP2A’s role in checkpoint stabilization and fork recovery is mediated through B55 alpha, consistent with literature implicating this subunit in regulation of WEE1/PKMYT1 and replication fidelity.^52–54^ Meanwhile, a distinct set of B55-independent activities likely underpin its interaction with core cyclin/CDK regulators (Figure 5I). Intriguingly, this bifurcation in regulatory logic may reflect a broader principle wherein multi-subunit complexes exhibit modular rewiring across environmental contexts to tailor their activity to specific cellular needs. Importantly, the trends observed from our GI maps were recapitulated in fluorescence competition experiments between all pairings of PPP2R1A/PPP2R2A and WEE1/PKMYT1/CDK2 (Figure S6).

By capturing these dynamics, our GI, eGI, and dGI maps open the door to systematic dissection of pleiotropic gene function across contexts. This not only facilitates clearer interpretation of complex biological factors like PP2A but also enables discovery of condition-dependent relationships that would be missed in single-condition screens. Expanding this framework to additional regulatory subunits of PP2A—and applying similar logic to other complexes—will further clarify how modular complexes are shaped by cellular context.

### The TIP60 complex displays extensively rewired GIs

The emergence of condition-specific GI clusters—such as TIP60 in ATRi, PP2A in etoposide, and glycolytic genes in glucose deprivation—highlights how cellular pathways can function as adaptive modules whose connectivity and influence expand or contract depending on the environmental context. These “coordinating ontologies” appear to orchestrate broader cellular responses, making them ideal entry points for understanding how specific perturbations trigger systemic adaptations. Among these, the TIP60 complex stood out due to the breadth and diversity of its ATRi-specific interaction profile, which mirrored its multifaceted roles documented in the literature.^43–46,55^

**Figure 6.**
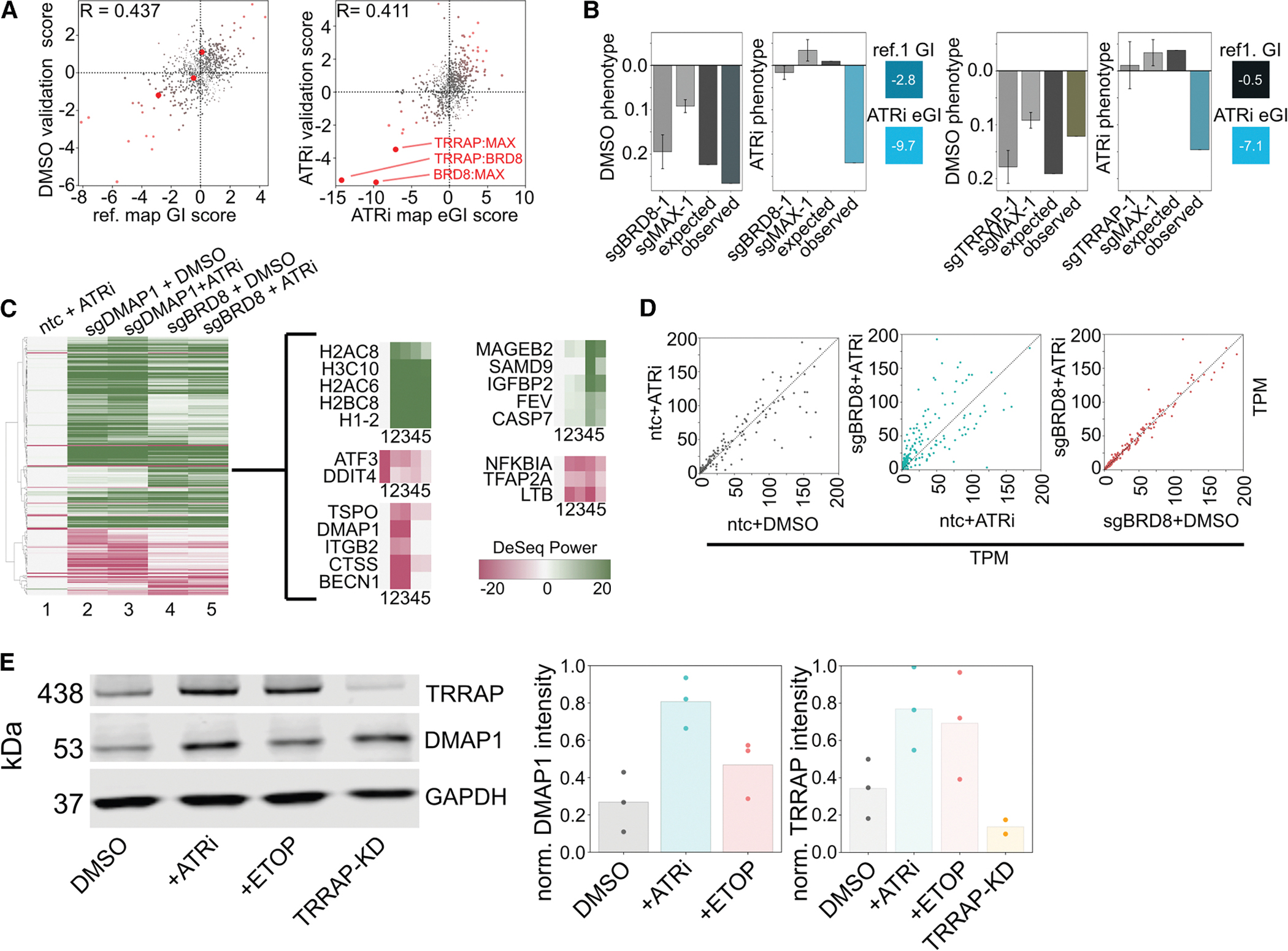
Investigation of TIP60 regulation and function (A) Scatterplots of GI scores from the initial reference (left) or ATRi eGI map (right) vs. scores from a validation matrix under the same respective conditions. Designed validations (e.g., TRRAP-MAX) are highlighted in red. (B) Phenotypic basis of two conditional GIs highlighted in (A); error bars denote standard deviation. (C) Heatmap of top 229 differentially expressed genes (ranked by |LFC × –log_10_(p)|) from RNA-seq in K562 across five comparisons vs. ntc + DMSO control. Red, depletion; green, upregulation. Genes clustered by Pearson correlation. Right: selected gene sets with coordinated expression patterns. Labels 1–5 mark comparisons. (D) Scatterplot of TPM values for all genes across three RNA-seq comparisons. (E) Left: western blot for TRRAP and DMAP1 in K562 cells treated with DMSO, ATRi, etoposide, or TRRAP knockdown. Right: bar plot of normalized band intensities (GAPDH control), averaged over three experiments (two for TRRAP KD).

To further investigate, we experimentally validated a subset of these condition-dependent interactions using both fluorescence-based competition assays and a focused GI matrix. Across both orthogonal approaches, we observed strong concordance with our initial screen, reinforcing the specificity and reproducibility of these interactions (Figures 6A, 6B, S3A, S3B, S3F, and S3H).

TIP60 is best known as a nuclear-localized HAT complex, central to chromatin remodeling and transcriptional regulation. While our data confirms interactions with numerous nuclear regulatory factors, a substantial proportion of high-confidence GI in ATRi occurs with genes encoding proteins localized outside the nucleus, possibly via indirect mechanisms, such as TIP60 broadly reshaping the transcriptome in response to ATR inhibition, thus affecting many downstream processes.

To test this, we conducted bulk RNA sequencing (RNA-seq) following genetic and chemical perturbation of TIP60 and ATR pathways (Figures S7A and S7B). We found that genes included in our GI library were broadly not differentially expressed following ATRi treatment or perturbation of TIP60 (Figures S7C–S7G). Furthermore, there was no correlation between high-magnitude dGI scores in the ATRi condition and differential expression of those same genes (Figure S7H). These results indicate that the widespread changes in GI observed are not simply a reflection of ATRi- or TIP60-KD-induced transcriptional shifts, supporting the idea that TIP60’s role in ATRi-specific interaction profiles is mediated through post-transcriptional mechanisms or protein-level dynamics.

Knockdown of TIP60 complex members had a substantial transcriptional footprint. In particular, BRD8 and DMAP1 knockdown led to widespread differential expression, exceeding the magnitude of change induced by ATR inhibition alone. Shared expression changes between these perturbations include the upregulation of nucleosome-encoding genes and repression of stress response regulators such as NFKBIA, suggesting a conserved role for TIP60 in maintaining chromatin and transcriptional homeostasis (Figure 6C). Notably, some responses were subunit-specific: for instance, DMAP1 knockdown uniquely downregulated BECN1, implicating a specific role in autophagy and apoptosis regulation.

A striking observation was the near-complete lack of differential gene expression between TIP60-depleted cells treated with DMSO vs. ATRi. This suggests that TIP60 loss renders cells largely refractory to the transcriptional effects of ATR inhibition. This phenomenon held even when comparing raw transcript abundances (TPM), where correlation was nearly perfect and variance minimal between treated and untreated TIP60 KD samples (Figure 6D). By contrast, TPM distributions differed substantially when comparing WT cells with or without ATRi treatment, further underscoring TIP60’s dominant influence over the transcriptional response landscape.

To probe whether ATRi might regulate TIP60 at the protein level rather than through transcriptional changes, we assessed protein abundance of complex components TRRAP and DMAP1 via western blot. Both proteins were significantly upregulated upon treatment with ATRi and etoposide (Figure 6E), suggesting TIP60 complex abundance is responsive to cellular stress through post-transcriptional or translational control. Intriguingly, TRRAP knockdown led to compensatory upregulation of DMAP1, consistent with homeostatic interdependence often observed in obligate multiprotein complexes. Finally, we observed TRRAP and DMAP1 are localized to the nuclear compartment under all treatment conditions (DMSO, ATRi, and etoposide), validating that the functionality of the complex is likely isolated to its effects on genome organization in the nucleus (Figure S7I).

Collectively, our data suggest that the TIP60 complex is a central node in coordinating cellular homeostasis. Its members can operate interdependently, with the loss of any single component triggering broad changes in transcription and interaction profiles. The GI observed in ATRi reflects a TIP60-mediated sensing mechanism that integrates metabolic, regulatory, and stress signals into a coordinated transcriptional response. Moreover, TIP60 activity appears to dominate over ATR signaling in shaping the gene expression landscape, suggesting a hierarchical organization in which TIP60 buffers or overrides ATRi-induced responses. Future studies incorporating additional components of the TIP60 complex and extending to other stress contexts will further illuminate its dynamic and pleiotropic contributions to cell fate regulation.

### Integrated analysis of GI and eGI matrix clusters enables higher-level analysis of ontology-level rewiring

Initial inspection of the five GI and eGI maps revealed that ontologically coherent gene clusters were generally preserved across conditions, exhibiting few changes in membership. To more rigorously assess both intra- and inter-complex relationships across all environmental contexts, we sought to generate a consensus clustering that would unify the breadth of interaction profiles observed in our study and facilitate robust cross-condition comparisons.

**Figure 7.**
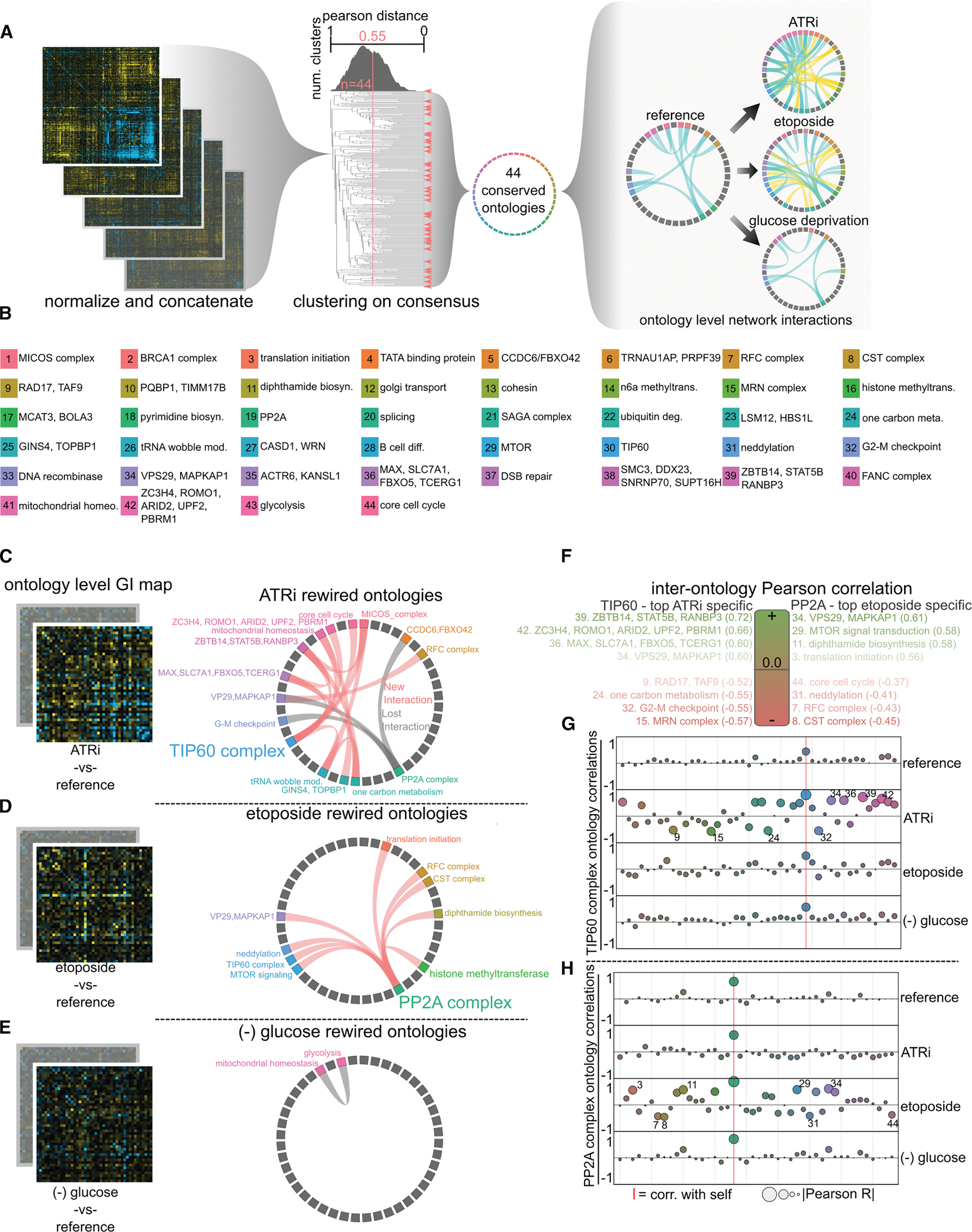
Ontology-level analysis of environmentally induced functional rewiring (A) Strategy for identifying conserved ontologies via consensus clustering of two reference GI and three eGI maps. Each map was normalized and concatenated to form a consensus matrix, then clustered by average pairwise Pearson correlation. Applying a Pearson distance threshold yields 44 conserved gene clusters (see STAR Methods). These clusters serve as the basis for ontology-level GI maps. (B) Description and color-coding of the 44 conserved clusters. Clusters lacking enriched GO terms or known complex identity are described by constituent genes. (C–E) Environmental rewiring of inter-ontology interactions. Pink edges indicate interactions gained under treatment; gray edges represent interactions lost relative to the reference. (F) The top four most and least correlated ontologies to the TIP60 cluster under ATRi and to PP2A under etoposide. (G and H) Correlation of TIP60 (G) or PP2A (H) with all conserved clusters across all GI/eGI maps.

To construct this consensus view, we first independently normalized each of the five interaction maps by their respective standard deviations. The normalized maps were then concatenated along one axis to form a composite *n* × *5n* matrix. We applied hierarchical clustering to the short axis of this matrix using average linkage of the Pearson correlation. The resulting dendrogram served as a universal clustering framework that could be retroactively applied to each individual GI or eGI map to provide a standardized ontology space for comparison (Figure 7A).

To validate that this approach preserved underlying biological signals, we tested the consensus clustering at the sgRNA level. Because most genes in our dataset are represented by two independent sgRNAs, the correlation between their interaction profiles serves as a metric of clustering fidelity. We found that the median Pearson correlation between same-gene sgRNAs in the consensus matrix was 0.37—comparable to or exceeding the correlations observed in individual maps, with the exception of the ATRi, eGI and sgRNA matrix, which showed slightly higher internal concordance (Figures S8A–S8F).

Applying the consensus clustering to gene-level GI maps enabled the identification of 44 conserved gene clusters. To define these clusters, we selected a Pearson correlation threshold of 0.55 from the consensus distance matrix, a cutoff that maximized cluster number while minimizing the merging of functionally distinct subclusters (Figure S8G). Each cluster’s gene set was manually tested against the MSigDB database to identify any enriched ontology terms. Twenty-six of the 44 clusters showed strong enrichment (*p* ≤ 1.0e–5) for known GO terms related to biological processes, molecular functions, or cellular components (Figure 7B; Table S11). An additional six clusters, while not enriched for any specific GO term, contained genes previously linked to common functional modules or protein complexes. The remaining 12 clusters lacked any known ontological annotation, making them particularly interesting as potentially undefined functional units.

Clusters lacking ontology assignment often grouped genes whose products had shared cellular localizations and superficially similar functions but no formal relationship. Among these gene sets are *PRPF39* and *TRNAU1AP*, both predicted to have RNA processing-related domains and nuclear localization but more established in tRNA maturation and splicing activities, respectively. Similarly, *ACTR6* and *KANSL1* have no known association but are both annotated as having histone modification activities and cluster reliably across our datasets (Table S11).

To examine how functional modules are rewired across environments, we calculated the average pairwise GI or eGI score within each of the 44 consensus clusters in all five conditions, producing compressed ontology-level matrices. Differential matrices were then generated by subtracting each reference matrix from its corresponding environmental condition, revealing broad changes in cluster-cluster interactions. These rewiring patterns offer a higher-level view of functional network plasticity and highlight the emergence of coordinating ontologies such as the TIP60 complex in ATRi, PP2A in etoposide, and glycolysis in glucose deprivation. Circos plots of these differential matrices provide an intuitive visualization of interaction rewiring and ontology crosstalk across conditions (Figures 7C–7E).

We further evaluated the conservation of clustering across maps by comparing pairwise gene correlation profiles between conditions (Figure S8H). As expected, the two reference maps were most highly correlated. Environmental maps exhibited more variable correlation patterns, with the ATRi and etoposide conditions displaying broadening distributions of both positive and negative correlations. This expansion suggests that genotoxic stress may simultaneously enhance cooperative interactions across distant ontologies while also decoupling previously stable genetic relationships. Such plasticity may reflect the need for cells to reconfigure regulatory networks in response to genome-destabilizing conditions.

Interestingly, while intra-complex correlations were generally conserved across conditions, inter-ontology relationships were far more variable. This pattern was disrupted most prominently in glucose deprivation, where the mitochondrial supercluster showed diminished internal coherence, likely reflecting metabolic reprogramming under nutrient stress. Overall, these observations suggest that while core complexes maintain internal consistency across conditions, their external connectivity to other biological processes is highly environment dependent.

Finally, we leveraged the consensus ontology framework to interrogate how biological modules coordinate or decouple their activity in different contexts. We developed an approach to compute the correlation between a query ontology and all other ontology-level clusters across each of the five conditions. This method enables tracking of ontological behavior and coordination as a function of environmental context. To illustrate this framework, we focused on two ontologies that showed dramatic shifts in coordination: the TIP60 complex in ATRi and the PP2A complex in etoposide (Figures 7F–7H). In the ATRi condition, TIP60 gained strong positive correlations with ontologies lacking GO enrichment but enriched for gene regulatory elements, including zinc-finger proteins and transcription factors. Simultaneously, TIP60 negatively correlated with cell-cycle checkpoint and DDR genes, consistent with a role in promoting transcriptional programs that support growth and metabolic adaptation over stress mitigation. By contrast, PP2A coordination in etoposide revealed a different rewiring logic, with strengthened ties to DNA repair and chromatin remodeling factors.

Together, these results establish a compelling new analytic paradigm for studying gene interaction networks at the ontology level across variable environments. We show that environmental stressors do not merely perturb single genes but restructure entire modules of genetic connectivity in a functionally meaningful manner.

## DISCUSSION

In summary, this study presents the first systematic, multi-condition measurement of context-specific GI in human cells. Using a scalable GxGxE mapping platform and innovative analytic approaches, we uncover the dynamic rewiring of gene networks in response to cell-cycle inhibition, genotoxic damage, and nutrient deprivation. We find that intra-complex interactions are largely conserved, whereas inter-complex relationships are extensively rewired, revealing new context-dependent genetic dependencies. Our findings demonstrate that gene function is highly plastic and contextually defined, challenging the classical notion of static gene roles and emphasizing the need for environmental context in functional genomics.

This study makes great effort to establish an intellectual and experimental framework for how to interpret changes in GI phenotypes across conditions by using environmental perturbations in the form of small molecule inhibition of critical cellular kinases, induction of DNA damage, and nutrient deprivation.

### Limitations of the study

It remains to be determined the degree to which comparison across cell types reveals similar or different dGIs. Our framework for measuring dGIs has only been evaluated in one cell type. Furthermore, the K562 cell line used in this study is deficient in p53 signaling. As many of the genetic relationships we investigate here are between genes implicated in DNA repair and cell-cycle regulation, our reported phenotypes are likely to be at least somewhat different when measured in a cell line with active p53 checkpoint signaling.

## RESOURCE AVAILABILITY

### Lead contact

Requests for further information and resources should be directed to and will be fulfilled by the lead contact, Luke Gilbert (luke@arcinstitute.org).

### Materials availability

All unique/stable reagents generated in this study are available from the lead contact with a completed materials transfer agreement.

### Data and code availability

- Raw and processed sequencing data can be accessed at GEO accession number GEO: GSE312636 or https://app.box.com/s/nuwov4kgb55mqfrr7j5a216ot78f4uxn. Unprocessed western blot imaging data are available through Zenodo: 10.5281/zenodo.17968835.
- All original code has been deposited at https://github.com/bherken/herken2024_originalcode and is available through Zenodo: 10.5281/zenodo.17822522
- Any additional information required to reanalyze the data reported in this paper is available from the lead contact upon request.

## STAR★METHODS

### EXPERIMENTAL MODEL AND STUDY PARTICIPANT DETAILS

#### Mammalian cell line culture and lentivirus preparation

All mammalian cell lines were cultured at 37^◦^C with 5% CO2. K562 cells stably expressing dCas9-KRAB^21^ were grown in RPMI-1640 media with 25mM HEPES (GIBCO), supplemented with 10% FBS, 100units/mL Penicillin, 100μg/mL Streptomycin, and 292μg/mL Glutamine (GIBCO). During the nominating and GI screens, K562 were grown in shaker flasks in a dedicated cell culture shaker/incubator (INFORS) at 120rpm and the media these cells were grown in was further supplemented with 0.1% Pluronic surfactant (GIBCO). A549 cells stably expressing dCas9-KRAB were grown in F12K media supplemented with 10% FBS, 100units/mL Penicillin, 100μg/mL Streptomycin, 292μg/mL Glutamine (GIBCO), 1x MEM Non-Essential amino acids (GIBCO), and 1mM Sodium Pyruvate (GIBCO). HEK293T cells were grown in DMEM media supplemented with 4.5g/L D-Glucose, 110mg/L Sodium Pyruvate (GIBCO), 10% FBS, 100units/mL Penicillin, 100μg/mL Streptomycin, 292μg/mL Glutamine (GIBCO). Lentivirus was prepared from HEK293T cells using TransIT-LT1 transfection reagent (MIRUS) and standard three-vector packaging plasmids. 72hr post-transfection, viral supernatant is filtered with 0.45μm filters and frozen at −80°C.

### METHOD DETAILS

#### sgRNA expression vector and library design

All sgRNAs used in all experiments were derived from a previously described list of the top5 algorithmically determined CRISPRi guides for each protein-coding gene in the genome.^34^ This genome-scale CRISPRi library was used for the nominating CRISPRi ATRi screen.

To clone the library used for GI mapping: the two sgRNAs with the strongest phenotype from 293 genes in the nominating screen with a |ρ| score (see below) >0.2 and Mann-Whitney p-value < 0.02 were used as the basis for our focused GI library. To this list of 586 sgRNAs, 40 more were included targeting 20 genes with a known connection to a range of gene ontologies pertaining to the DNA damage response that had been validated in a previous study.^24^ Finally, 13 negative control sgRNAs were included bringing the total size of the library to 639 unique sgRNAs (Figure 2B; Table S2). Protospacers for this library were obtained in a pooled format commercially, subsequently cloned into two independent intermediate vectors, then cloned into the final dual-sgRNA expression vector in which every possible pairwise permutation of sgRNAs in the singles library is represented, as described previously.^24^ Through attrition inherent in cloning large sgRNA libraries, a small percentage (<1%) of sgRNA pairs were lost, resulting in a realized library size of 405,667 with 90% of all elements expressed at <16-fold variation (Figures S1E and S1F).

Dual-sgRNA vectors used in fluorescence competition validation experiments were cloned in the same expression vector as used in the GI library, in an arrayed format.

Single sgRNA vectors used in RNAseq experiments were cloned into the same vector used in the nominating CRISPRi screen, in an arrayed format.

#### Transduction and screening with CRISPRi libraries

Transduction and passaging of cells was performed in duplicate as independent biological replicates for all experiments. For nominating and GI screens, K562 cells stably expressing dCas9-KRAB were transduced with a lentiviral preparation of an sgRNA library using 8μg/mL polybrene with the goal of obtaining a transduced population representing at least 250-fold coverage of the library while maintaining an MOI below 0.3. This population of cells is grown and expanded continuously and dosed with puromycin at a concentration of 1μg/mL 48 and 72 hours post transduction to select for infected cells. The infected population was expanded until at least 4000-fold coverage of infected cells were available to begin the screen. At the initial timepoint, cell pellets with 1000-fold coverage of the library were frozen in 10% DMSO freezing media. This initial timepoint was taken at day six and day seven post transduction for the nominating screen and all GI screens respectively. Final timepoint samples for all screens were collected as 1000-fold coverage of the library.

For the nominating screen, samples were split to a concentration of 2.5e5 cells/mL every other day in fresh media. For the ATRi treated arm of the screen, on days zero and seven the treated samples were dosed with 750nM AZD6738, and the untreated samples were dosed with an equivalent volume of DMSO. Fifteen days after the start of the experiment, once the cumulative difference in population doublings between the treated and untreated samples had approximately reached five, the final timepoint was taken.

For the ATRi GI screen, samples were split every day to a concentration of 5e5 cell/mL in fresh media. On days zero and seven of the screen, the treated sample was dosed with 1.25μM AZD6738, and the untreated samples were dosed with an equivalent volume of DMSO. Final timepoint samples were collected on day nine after the cumulative difference in population doublings between the treated and untreated populations exceeded 4.5 in at least one replicate.

For the etoposide GI screen, on day zero, the treated samples were dosed with 500nM etoposide and untreated samples with an equivalent volume of DMSO. Media was washed out and replaced with fresh untreated media 24 hours later. Three days after the start of the experiment, cells were again dosed with 500nM etoposide. On subsequent days cell populations were daily split back to a concentration of 5e5 cells/mL of media but without media washout, allowing a longer exposure time for the etoposide sample. Final timepoint samples were taken at day nine once differences in cumulative population doublings between the etoposide and control populations had reached at least 4.0 in at least one replicate.

For the glucose deprivation GI screen, on day zero, the treated samples were resuspended in RPMI media without glucose. Treated cells were grown in this type of media for the length of the experiment. Every day all samples were analyzed and split to a concentration of 5e5 cells/mL in fresh media. Final timepoint samples were collected on day nine once differences in cumulative population doublings between the glucose deprived and control populations had reached 2.33 in at least one replicate.

For both the nominating screen and all GI screens, genomic DNA was extracted from T0 and final timepoint cell pellets using a Macherey-Nagel Blood XL cleanup kit. Integrated sgRNA loci were amplified from gDNA using PCR to append adapters for NGS, then submitted for sequencing on an Illumina Hiseq4000 or NovaSeq platform, for the nominating and GI screens respectively. For GI libraries, a hamming distance of one is used when mapping reads to our reference library, to allow for minor errors in sequencing to not be excluded from analysis. Custom sequencing primers were used for both nominating screen and GI maps as described previously,^24,34^ see key resources table for details.

#### Fluorescence competition assays

K562 or A549 cells are transduced with a lentiviral preparation of a single or dual sgRNA expression vector and expanded continuously for several days. At day five post transduction, cells are seeded at a concentration of 5.0e5 cells/mL and dosed with 1.25μM AZD6738, 500nM of etoposide or an equivalent volume of DMSO, constituting the d0 sample. Each condition is analyzed by flow cytometry everyday or every other day to determine the ratio of uninfected control cells to BFP+ transduced experimental cells before being reseeded at 5.0e5 cells/mL in fresh medium. This process is repeated until seven or eight days after the first dose or the transduced population falls below 1% of the total population. Samples are sometimes given a second dose of each drug if the total population’s viability is recovering from the initial dose and the transduced population still represents a significant portion of the total population.

All samples are processed by normalizing the final timepoint %BFP+ by their respective day 0 %BFP+ transduction efficiency, then log2 transforming these values to calculate the enrichment/depletion phenotypes. For every gene pair, a predicted phenotype is calculated by summing the individual gene phenotypes (sgRNA-ntc pair). The observed pair phenotype is compared against this predicted phenotype in order to determine if the gene pair is exhibiting non-additive behavior consistent with genetic interaction.

#### Propidium Iodide (PI) Staining of DNA content

Pure populations of cells transduced with an sgRNA targeting CCDC6, FBXO42, or ntc were grown in media treated with 1.25μM AZD6737 or an equivalent volume of DMSO for 24hr. Cells were washed and grown in fresh untreated media for another 24hr before harvesting for PI staining (total time post treatment = 48hr). To prepare for staining and flow cytometry, cells were washed in PBS (GIBCO) and cell pellets resuspended in ice-cold 70% EtOH while being vortexed. Fixation continued at −20°C for 1hr, then cells were washed in PBS and analyzed by flow cytometry to determine concentration. 5E5 fixed cells were spun down and resuspended in 500μL of PI/RNase staining solution (Invitrogen). Cells were stained for 30 minutes then analyzed by flow cytometry to determine DNA content distributions of each population.

#### RNA-seq

K562 cells are transduced with a lentiviral preparation of a single sgRNA expression vector targeting either BRD8, DMAP1, or a ntc in replicate. 48hr post-transduction cell populations are sorted to obtain a pure population of transduced cells and expanded continuously for several days. On day five post-transduction, cell populations are dosed with 1.5μM AZD6738 or an equivalent volume of DMSO. 48hr hours after dosing 1.0e6 cells are harvested from each sample. RNA is then extracted from cell pellets using a Zymo Direct-Zol kit. 350ng of RNA from each sample is used as the input for the Lexogen Quantseq FWD kit with UMI add-on. All steps of the kit are performed according to the manufacturer’s instructions.

#### Validation matrix library design and screening

A subset of 76 sgRNAs from the full GI library representing 38 genes (two guides per gene) were selected based on a criteria of low absolute eGI profile in the ATRi condition. To this pool, eight additional sgRNAs were added targeting four genes: TRRAP, BRD8, MAX, and MTOR. These sgRNA were chosen based on high interaction scores between them calculated in the ATRi condition. Six ntc guides were added to the pool for a total size of 90 sgRNAs. Every pairwise permutation of sgRNAs was cloned as described in “sgRNA expression vector and library design” above to yield a final library of ∼8000sgRNAs. The library was packaged into lentivirus and used to transduce K562 cells, which were then selected for, dosed with DMSO, ATRi, etoposide, and glucose deprived RPMI, then passaged and sequenced as described in “Transduction and screening with CRISPRi libraries” with the following modifications. All samples were passaged for 12 days. Etoposide and ATRi cells were dosed at 1.25μM and 750nM, respectively, a single time and without washout for 72hr starting at day 0, after which these samples were grown in normal RPMI. 2000-fold coverage of each library was processed and sequenced for each library. Data analysis was performed as described below in “Calculating sgRNA level growth phenotypes from CRISPRi screens” and “Calculating genetic interactions”.

#### Western Blots

K562 CRISPRi cells were cultured in RPMI with either 1.25μM of ATRi, 750nM of etoposide, or an equivalent volume of DMSO for 72hr. K562 CRISPRi cells transduced with a sgRNA from our GI library targeting TRRAP were similarly cultured in DMSO containing RPMI. Cells were washed in dPBS, cell pellets were weighed and then lysed with MPER (Thermo cat#78501) supplemented with a protease inhibitor (Thermo cat#A32955) at a ratio of 1mL buffer/100mg cells by thorough mixing on ice with occasional vortex for 10mins. Cell lysates were clarified by spinning at 4000xg for 15mins in a benchtop centrifuge. Lysate supernatants were transferred to a new tube, and protein content measured by Qubit Protein BR assay. Cell lysates were normalized by dilution with MPER based on protein content.

For fractionation experiments, K562 cell lysates treated as described above were processed using a Nuclear and Cytoplasmic Extraction Reagents kit (Thermo cat#78833) according to manufacturer instructions. Protein content for all samples and fractions determined by Qubit Protein BR assay and normalized using respective buffers.

Fluorescent compatible loading buffer (Thermo cat#LC2570) was added to each lysate then lysates were incubated at 70^◦^C for 10mins. 40, 20, and 10ug of each lysate (1:2 serial dilutions in MPER) from each condition (5μg of all samples for cell fractionation experiments) were loaded into a 3–8% Tris-Acetate NuPage gel (Thermo cat#EA0378) with 1x running buffer (Thermo cat#LA0041) and a high range protein ladder (Thermo cat#26625), and run at 2hr at 120V on ice. Gel was transferred to a 0.45μm nitrocellulose membrane (BioRad cat#1620115) using a full wet transfer and 1x Tris-Glycine transfer buffer (Thermo cat#28363) for 90mins at 100V on ice. Blot was washed in dPBS, then blocked in Blockout reagent (Rockland cat#MB-073) for 2hrs. Blot was then cut with a razor right above the 40kDa ladder and right above the 250kDa ladder. Blot fragments were incubated at 4°C overnight with primary antibodies against either DMAP1 (Abcam AB188407, dilution 1:1000), TRRAP (Abcam AB187166, dilution 1:1000), or GAPDH (CST2118L, dilution 1:20000) diluted in Blockout. Blots were washed with TBST 3×5mins then incubated with an anti-rabbit 800nm fluorescent secondary antibody (CST5151, dilution 1:10000 in TBST) at RT for 1hr. Blots were washed 3×5mins in TBST then imaged using a LI-COR fluorescence imaging system. Raw unprocessed western blot imaging data presented in this manuscript is available at 10.5281/zenodo.17968835.

### QUANTIFICATION AND STATISTICAL ANALYSIS

#### Calculating sgRNA level growth phenotypes from CRISPRi screens

Calculating primary sgRNA level growth phenotypes for the nominating CRISPRi screen, GI screens, and validation screens were done in the same way. First, each sgRNA construct within a sample is normalized by the total read counts for all sgRNAs in that sample, to adjust for differences in sequencing depth. Phenotypes in an untreated condition (gamma), or treated condition (tau) are calculated as:

γ=log2RFNFR0N0×1duτ=log2RTNTR0N0×1dt

where **R_F_**, **R_T_**, and **R_0_** are the number of reads for the query sgRNA construct in the final untreated, final treated, and initial timepoints respectively. **N_F_**, **N_T_**, and **N_0_** are the median number of reads among negative controls (ntc-ntc pairs in GI screens) in the final untreated, final treated, and initial timepoints, respectively. **d_u_** and **d_t_** are the cumulative number of cell population doublings in the experiment in the untreated and treated conditions, respectively.

Additionally, to calculate the guide normalized effect on cell viability in the treated condition, that

ρ=log2RTNTRFNF×1d₍u₎-d₍t₎

is used to threshold hits in the nominating screen (rho), we use:

In the GI screen, single sgRNAs with median read counts less than 35 in either orientation (either first or second) in the expression vector in any condition are removed from analysis in that condition. Additionally, a pseudocount of 10 is added to each sgRNA’s read count that passes this threshold. Single sgRNA level phenotypes are then determined by averaging the gamma or tau of each sgRNA paired with all thirteen negative controls in both orientations (26 independent measurements per sgRNA, 52 per gene).

#### GO term enrichment

The enrichr function of the gseapy module in python was used to determine GO term enrichment of gene sets from the nominating ATRi screen, GI/eGI maps, and RNAseq datasets. The gene sets used as a reference were “GO_Biological_Process_2023”, “GO_Cellular_Component_2023”, and “GO_Molecular_Function_2023”. GO terms were considered significantly enriched in a gene set if the -log10 adjusted p-value associated with the term was >=6. For the nominating screen, GO terms enrichment was run on the screen’s hit list in successive iterations, where after each iteration the genes found in the most enriched GO term were removed from the hit list used to calculate GO terms in the successive iterations. This process was repeated 10 times to determine the top 10 most enriched yet non-overlapping GO terms.

#### Calculating genetic interactions

A GI model is generated for each single query sgRNA by quadratic regression of the relationship between all single sgRNA phenotypes and every paired sgRNA phenotype in which the query sgRNA is present (Figures 2A and 2B). A genetic interaction for all query paired sgRNA constructs is then calculated based on the deviation of that pair’s phenotype from the model. These differences are then z-score normalized by the standard deviation of the negative control distribution for that guide (standard deviation of phenotypes in the population of query sgRNA-ntc pairs). GIs called for each unique pair of sgRNA are averaged across both orientations of that pair (first and second in the construct). Gene-level GIs are called by averaging all combinations of guides that target those genes.

#### Clustering genes by GI profile

GI and eGI matrices were clustered using the “clustermap” function of the seaborn package in Python with the “method” and “metric” variables assigned as “average” and “correlation” respectively. Ontology-level clustering as shown in Figures 3A and 3C is calculated by setting a Pearson distance threshold that maximizes formation of new clusters without collapsing existing clusters together. We qualitatively decided on a Pearson distance of 0.55 as a best fit for all five maps. Similarly, for “System” clusters, we decided on a Pearson distance of 0.8 to sufficiently connect genes to the point where they represented larger areas of biology. To generate the “consensus” clustering, the GI and eGI map were first normalized by their standard deviation, then concatenated to form a 304 by 1520 element matrix. This matrix was clustered using the same methodology as the five independent maps.

#### Enrichment of genetic interactions for STRING protein-protein interactions

The absolute value of all scores in a GI matrix are taken and every unique gene pair is binned according to its absolute GI score in a given condition. STRING PPI scores (‘escores’) are taken for all individual genes in the map using the STRING API. Gene pairs in each bin are tested against the list of STRING PPIs and the average PPI score in each bin is taken (no interaction detected is measured as 0), and normalized by the average PPI score for all unique gene pairs in our full library to determine enrichment over background. Chi square analysis uses the same bins and assigns categorical values (interacting/not-interacting) based on any detected STRING PPI.

#### Differential expression analysis of RNA-seq data

Differential expression of genes calculated from RNAseq data was analyzed using the DeSeq2 package in R. Genes with fewer than 10 reads across all replicates and conditions were removed from the analysis as they are assumed to not be expressed in either K562 cells. Differential expression log-fold change and associated p-values were calculated for the five treated conditions (ntc+ATRi, sgBRD8+DMSO, sgBRD8+ATRi, sgDMAP1+DMSO, and sgDMAP1+ATRi) against the reference control (ntc+DMSO) experiment. Power score is reported as the product of the differential expression LFC score and its associated -log10(p-value).

#### Western blot quantification

Enrichment of western blot target signal was determined from three independent experiments and quantified in Fiji. We used the 20ug input sample for all lysates and experiments as the signal identified for all targets in these samples was found to be neither undetectable nor oversaturated. Band intensity for each target (TRRAP, DMAP1, GAPDH) was normalized by subtracting background noise in each lane. Then the background normalized TRRAP and DMAP1 intensities were normalized by the GAPDH intensity in each corresponding lane. The background and input normalized TRRAP and DMAP1 intensities were averaged across the three experiments (two experiments for the TRRAP-KD lysate) to create the bar plot shown in Figure 6E.

## Supplementary Material

MMC12

MMC3

MMC9

MMC6

MMC10

MMC11

MMC2

MMC1

MMC8

MMC4

MMC7

MMC5

SUPPLEMENTAL INFORMATION

Supplemental information can be found online at https://doi.org/10.1016/j.molcel.2026.01.025.

## Figures and Tables

**KEY RESOURCES TABLE:** 

REAGENT or RESOURCE	SOURCE	IDENTIFIER
Antibodies		
Anti-DMAP Antibody	Abcam	Cat#: AB188407
Anti-TRRAP Antibody	Abcam	Cat#: AB187166
Anti-GAPDH Antibody	Cell Signaling Technology	Cat#: 2118; RRID: AB_561053
Anti-rabbit 800nm fluorescent secondary antibody	Cell Signaling Technology	Cat#: 5151; RRID: AB_10697505
Chemicals, peptides, and recombinant proteins		
AZD6738	Selleckchem	Cat#:S7693
Etoposide	Selleckchem	Cat#:S1225
Deposited data		
Fastqs, counts table, references for genome-scale and GI CRISPRi screens and RNAseq	this paper	https://app.box.com/s/nuwov4kgb55mqfrr7j5a216ot78f4uxn/folder/294064137354
Raw and processed sequencing data	this paper	GEO accession: GSE312636
Raw Western Blot imaging data	this paper	Zenodo: 10.5281/zenodo.17968835
Experimental models: Cell lines		
K562 CRISPRi	Gilbert et al.^34^	N/A
Oligonucleotides		
sgRNA sequences for CRISPRi perturbations	this paper	See Table S2
GI 5^′^ Library prep primer (N denotes sample index), Sequence:aatgatacggcgaccaccgaGATCTACACNNNNNNcagcacaaaaggaaactcacc	Horlbeck et al. 2018^24^	N/A
GI 3^′^ Library prep primer, Sequence: caagcagaagacggcatacgaGATggcggtaatacggttatcca	Horlbeck et al. 2018^24^	N/A
Illumina GI sequencing primer R1,Sequence: tgttttgagactataaGtatcccttggagaaCCAcctTGTTGG	Horlbeck et al. 2018^24^	N/A
Illumina GI sequencing primer R2,Sequence: cgatttcttggctttatatatcttgTGGAAAGCCAcctTGTTGG	Horlbeck et al. 2018^24^	N/A
Illumina GI sequencing primer I1,Sequence: aacacacaattactttacagttagggtgagtttccttttgtgctg	Horlbeck et al. 2018^24^	N/A
Recombinant DNA		
pLG_GI2	Horlbeck et al. 2018^24^	Addgene Plasmid #111593
pLG_GI3	Horlbeck et al. 2018^24^	Addgene Plasmid #111594
pLG_GI4	Horlbeck et al. 2018^24^	Addgene Plasmid #111595
Software and algorithms		
GI calling, analysis, and downstream processing scripts	this paper	https://github.com/bherken/herken2024_originalcode
GI calling, analysis, and downstream processing scripts	this paper	Zenodo: 10.5281/zenodo.17822522

## Highlights

- Human genetic interaction maps of response to three environmental perturbations
- Presents a framework for using genetic interactions to map environmental rewiring
- Environmental perturbations promote widespread rewiring of genetic interactions
- ATR inhibition vastly alters genetic interactions among TIP60 and PP2A complex components
