# Supplementary material for: Large-scale mapping of environmental-genetic interactions illustrates the dynamic nature of cell-cycle and DNA repair regulation
Source: Mol Cell. Author manuscript; Available in PMC 2026 Apr 10. (PMC13068139; doi:10.1016/j.molcel.2026.01.025)
Supplement: MMC1 [file NIHMS2148368-supplement-MMC1.pdf]

Molecular Cell, Volume *86*

Supplemental information

Large-scale mapping of environmental-genetic interactions illustrates the dynamic nature of cell-cycle and DNA repair regulation

Benjamin W. Herken, Garrett T. Wong, Anna Mäkiniemi, Emma Lundberg, Thomas M. Norman, and Luke A. Gilbert

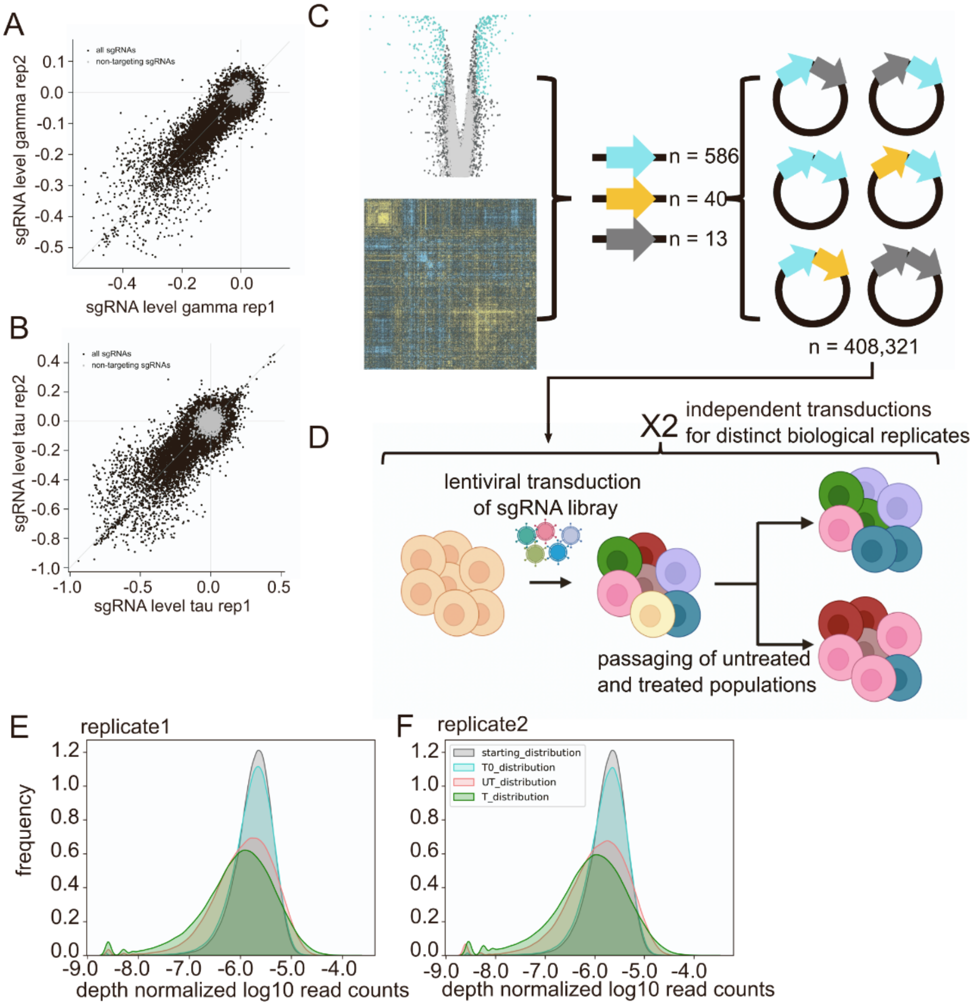

Figure S1. Quality control for nominating screen and dual-sgRNA library cloning. Related to Figure 1.

(A&B) sgRNA-level phenotype scatterplots from the control (A) and ATRi-treated (B) arms of the genome-scale CRISPRi nominating screen (γ and τ scores, respectively; see Methods). Gene-targeting sgRNAs are shown in black; non-targeting controls in light gray. **(C)** Schematic of dual-sgRNA GI library design and inclusion criteria. Cyan arrows: ATRi-specific validated sgRNAs from the nominating screen. Yellow arrows: sgRNAs from prior studies[24] related to DNA repair/cell cycle. Gray arrows: non-targeting controls with no phenotype in the nominating screen. **(D)** Overview of K562 screening strategy for both the nominating and GI screens (see Methods). **(E&F)** Kernel density plots of mapped dual-sgRNA read counts across four conditions for two replicates: pre-transduction, timepoint zero, final control, and final ATRi-treated (gray, blue, red, and green, respectively). Read counts normalized to total reads per condition.

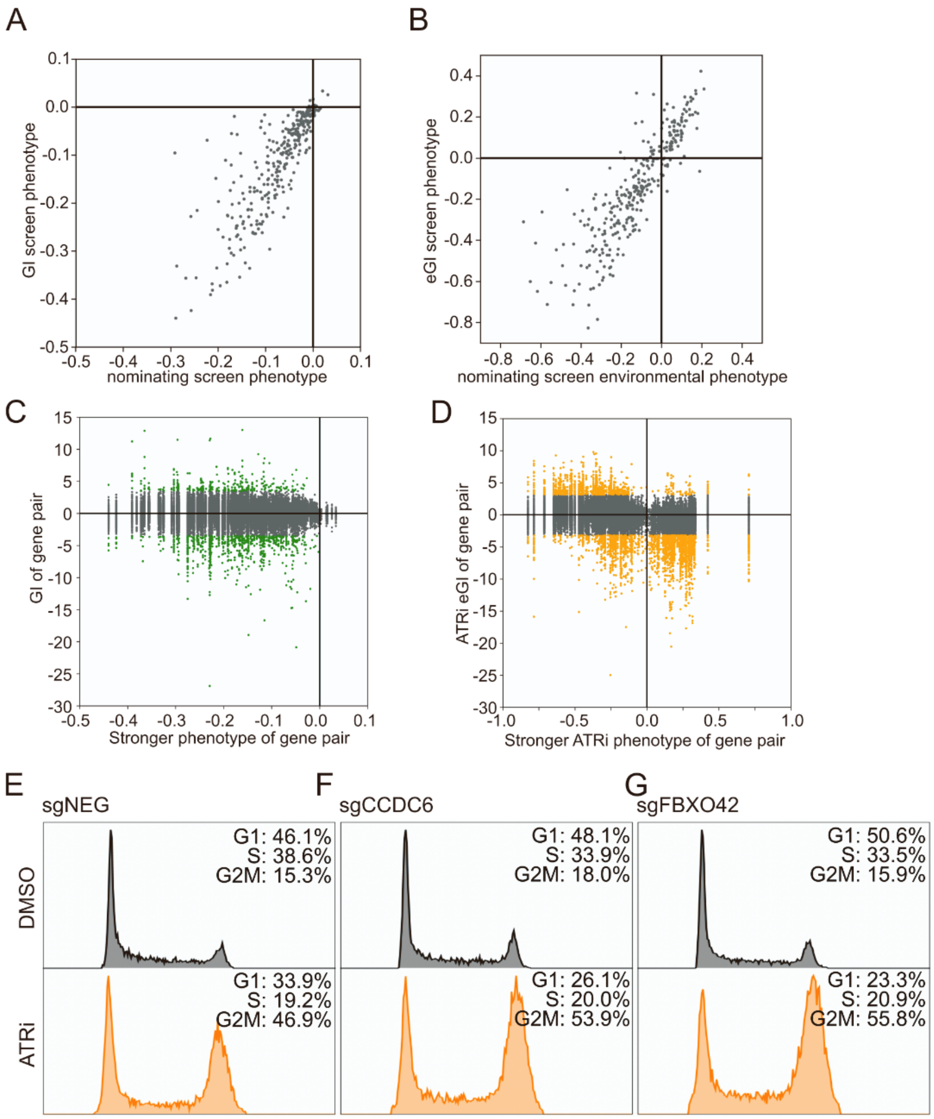

Figure S2. GI map quality control and phenotype validation. Related to Figure 2.
(A&B) Gene-level phenotypes (γ and τ) from the nominating CRISPRi screen compared to matched phenotypes in the GI (A) and eGI (B) screens. **(C&D)** Scatterplots of the stronger phenotype (farthest from zero) from each sgRNA pair under control (C) or ATRi-treated (D) conditions versus the calculated GI or eGI score. Gene pairs with significant interactions are highlighted in green (GI) or orange (eGI). **(E–G)** DNA content analysis by propidium iodide staining of cells expressing non-targeting sgRNA (E), sgCCDC6 (F), or sgFBXO42 (G) under DMSO or ATRi treatment.

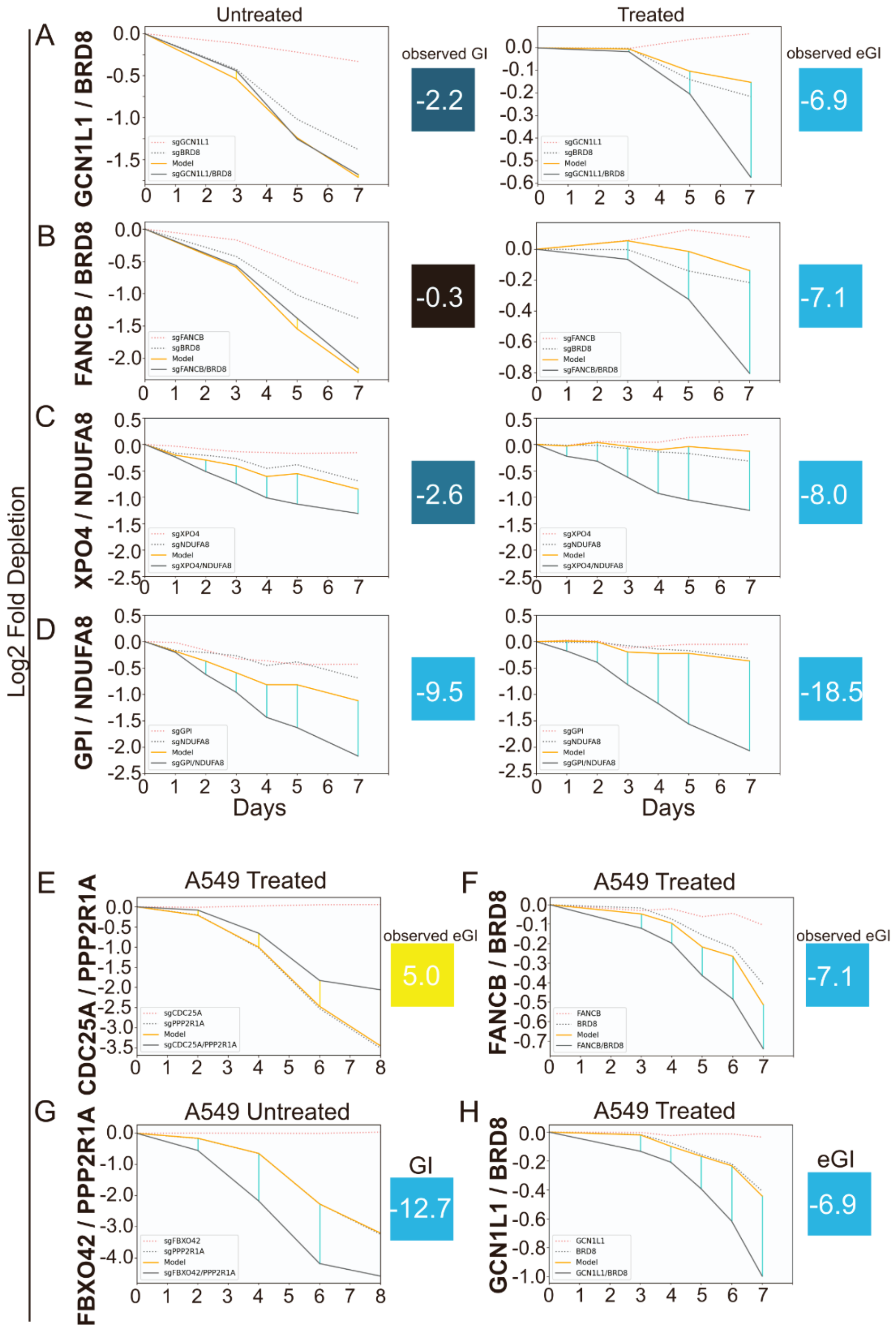

Figure S3. Fluorescence competition assays. Related to Figure2.

(A–D) Validation of environmentally conditional interactions. Dotted lines represent depletion phenotypes from single gene perturbations over time. Modeled paired phenotypes are shown in orange; observed paired phenotypes in gray. Interaction scores are visualized as vertical lines between modeled and

observed curves at each timepoint—colored blue (negative) or yellow (positive). GI and eGI values to the right reflect corresponding interaction scores from the maps. **(E–H)** Validation of interactions in A549 cells. Plot format matches that of panels A–D.

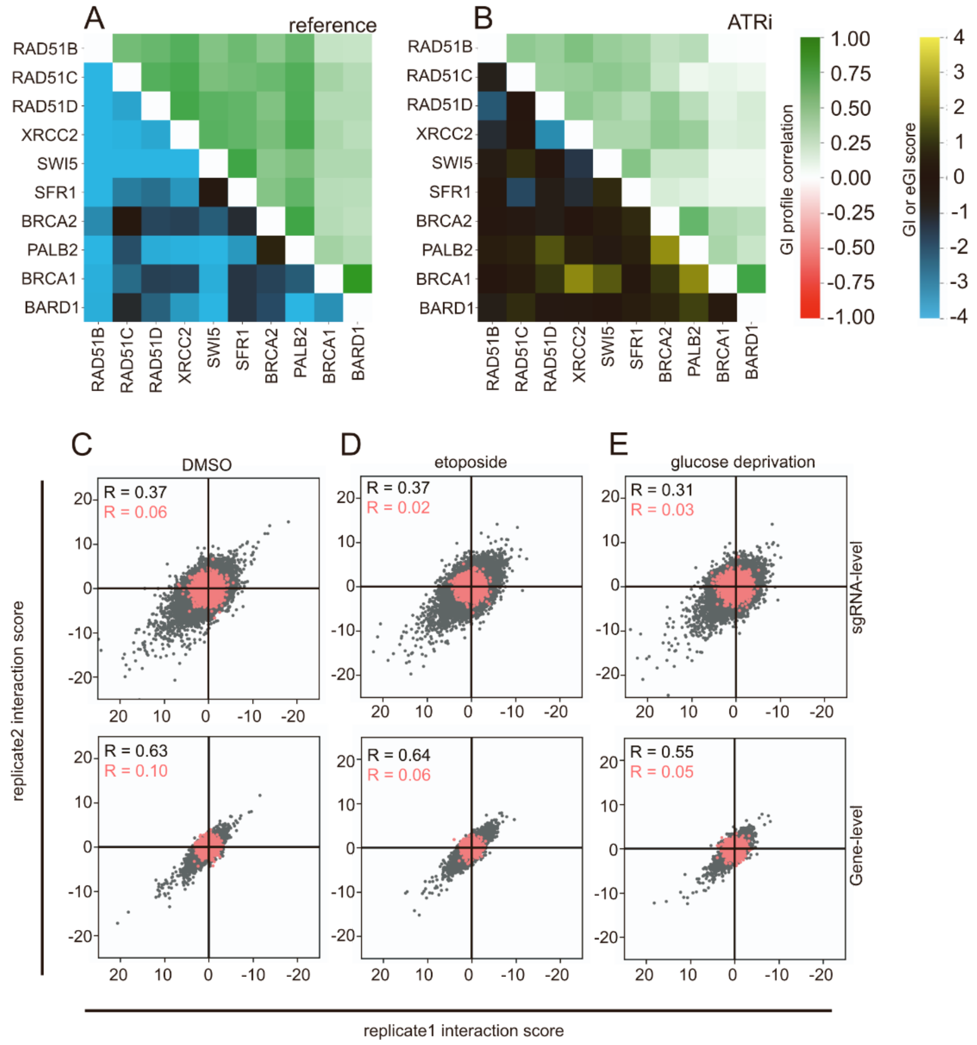

Figure S4. Conservation of clustering in differential interaction maps and replicate quality control of GI and eGI experiments. Related to Figure 3 & Figure 5.
(A&B) Subset of DNA repair genes from the reference GI (A) and ATRi eGI (B) maps. Lower triangle displays genetic interaction scores; upper triangle shows Pearson correlation between gene GI/eGI profiles. **(C–E)** Scatterplots of sgRNA-level (top row) and gene-level (bottom row) interaction scores for DMSO (C), etoposide (D), and glucose deprivation (E) maps. Pairs with non-targeting controls are highlighted in red. Pearson R values shown in top left of each plot.

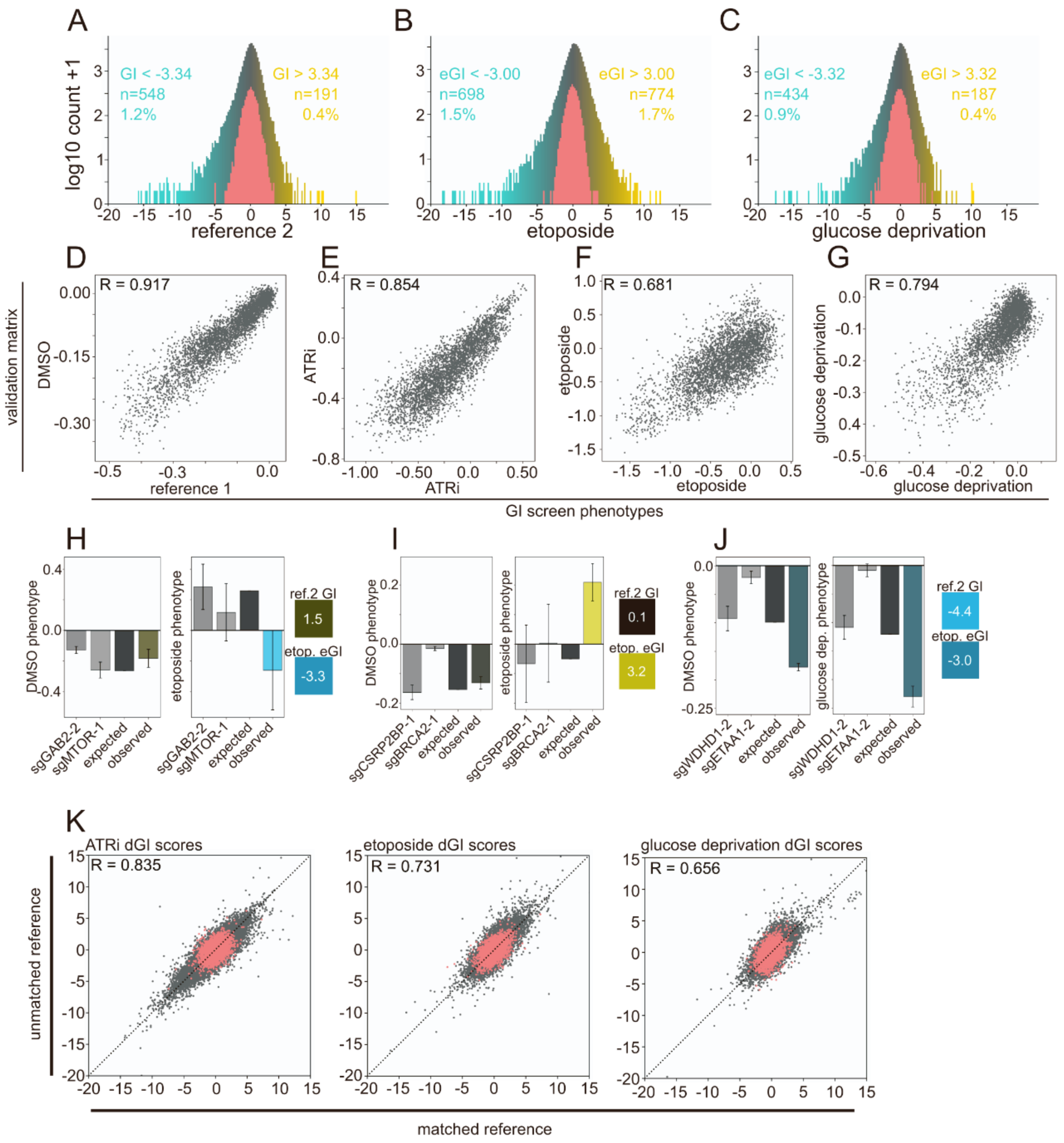

Figure S5. Interaction distributions and validation matrix analysis. Related to Figure 4 & Figure 5. **(A–C)** Distributions of interaction scores for DMSO (A), etoposide (B), and glucose deprivation (C) GI/eGI maps. Thresholds, number and percentage of significant interactions are annotated on both wings. Blue and yellow highlight negative and positive interactions, respectively; red highlights gene–ntc pairs. **(D–G)** Scatterplots comparing primary growth phenotypes (growth normalized log2 enrichment, see methods) from each GI/eGI map (x-axis) to those from the validation matrix for overlapping gene pairs. **(H–J)** Growth phenotypes from the validation matrix for selected single and paired gene perturbations under DMSO and treated conditions. Bars shaded by deviation from expected. **(K)** Scatterplots of dGI scores for each environmental condition calculated using matched (x-axis) or alternate (y-axis) references. Gene–ntc interactions are in red. Pearson R values inset.

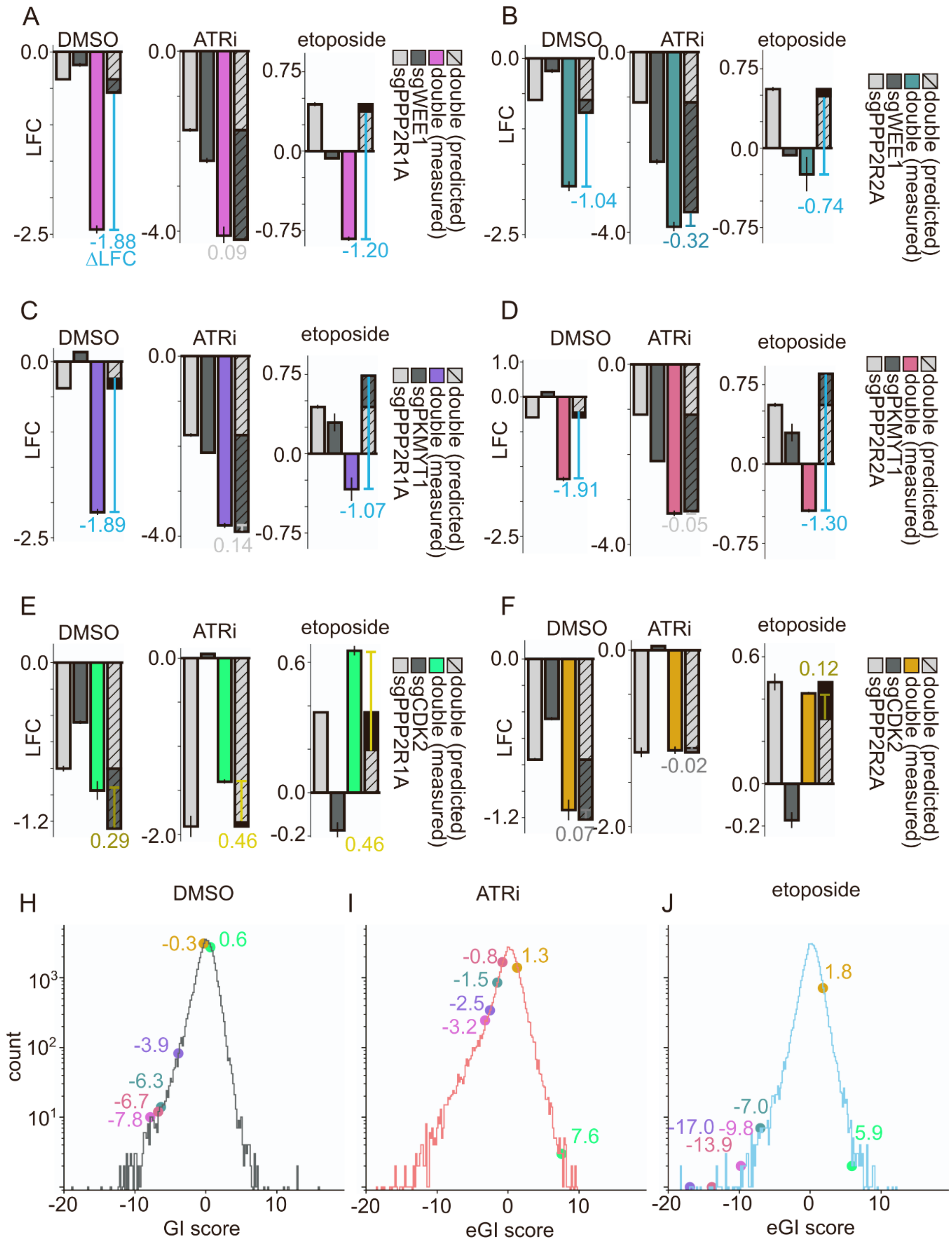

Figure S6. Validation of PP2A conditional interactions. Related to Figure 5.

(A-F) Fluorescence competition validation assays reporting log2 fold changes for all relationships between PPP2R1A/PPP2R2A and WEE1/PKMYT1/CDK2 in untreated, ATRi, and etoposide treated conditions. **(H-J)** Distributions of GI/eGI scores in the first reference, ATRi, and etoposide genetic interaction maps. All interactions tested in A-F are highlighted according to colors used in those figures with the associated scores called in each map.

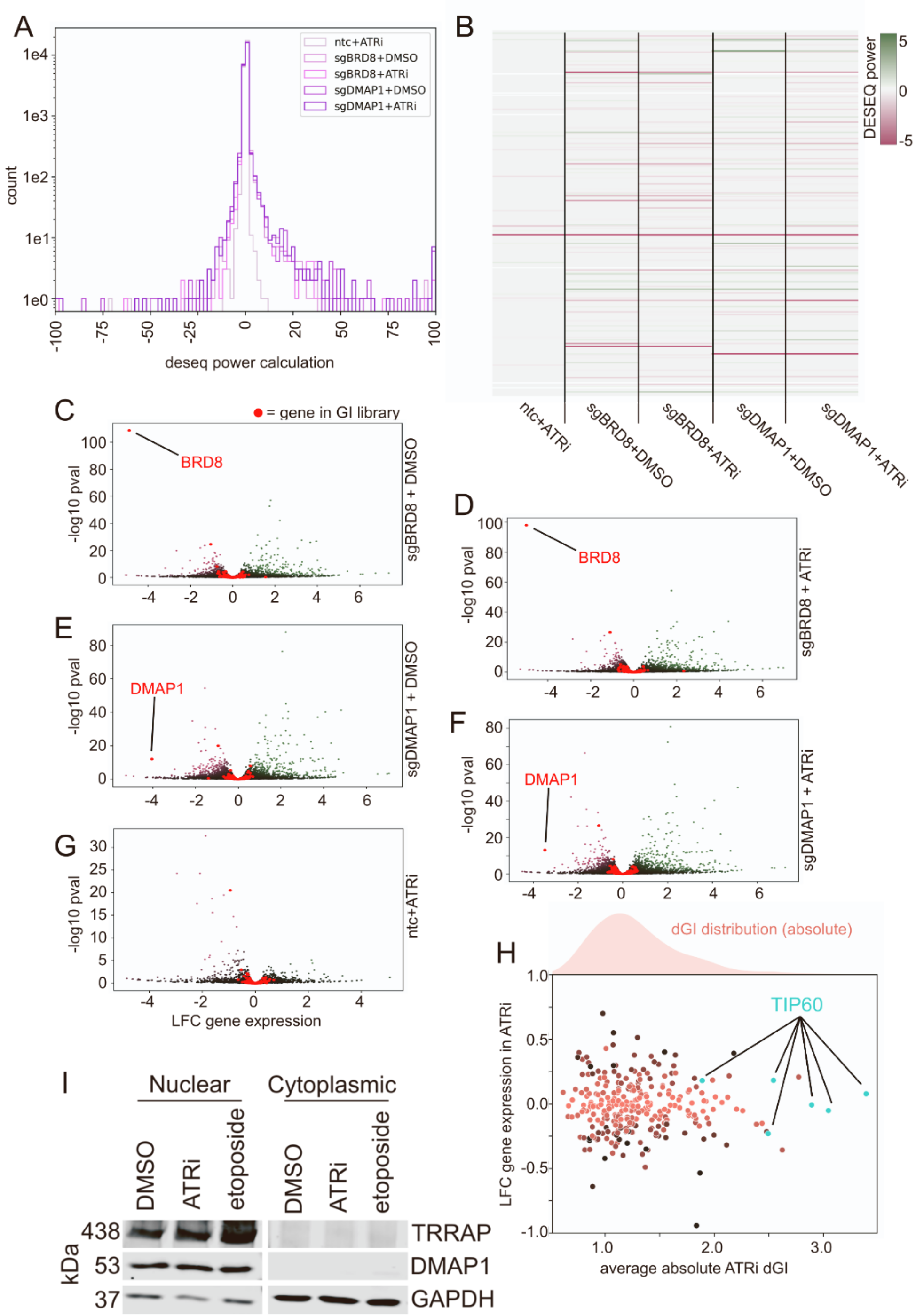

Figure S7. Bulk RNAseq analysis of TIP60 and ATRi effects on gene expression. Related to Figure 6.

(A) Histogram of DESeq2 power values (LFC × $-\log_{10}$(p-value)) for five experimental comparisons relative to ntc+DMSO. **(B)** Heatmap of DESeq2 power values for genes in the GI library. **(C–G)** Volcano plots of differential expression (LFC vs. $-\log_{10}$(p-value)) for each comparison. GI library genes are in red; other genes are shaded by power (LFC × $-\log_{10}$(p)). **(H)** Comparison of average absolute ATRi differential GI (dGI) scores to LFC gene expression changes in the ntc+ATRi comparison. Histogram

above shows distribution of absolute dGIs (pink). TIP60 genes are in light blue; genes with more significant expression changes are in darker pink. **(I)** Nuclear/cytoplasmic fractionated K562 cell lysate analyzed by western blot for TRRAP, DMAP1, and GAPDH.

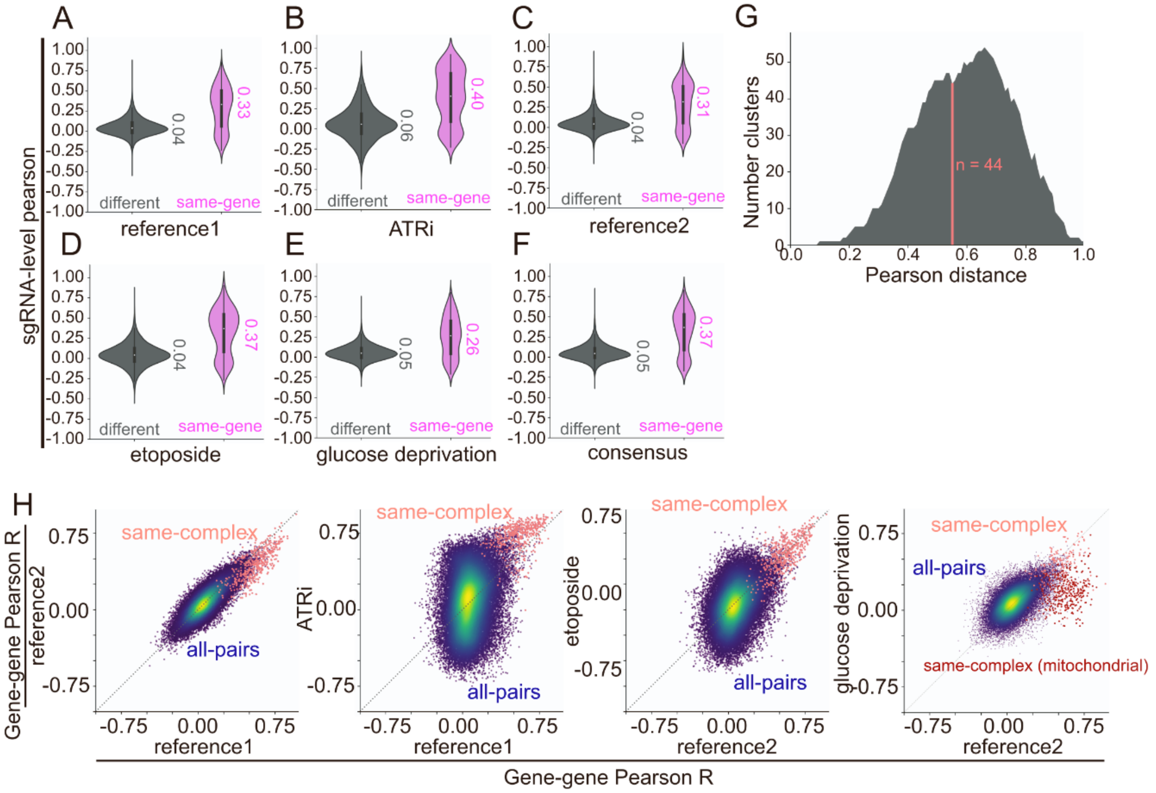

Figure S8. Consensus clustering of GI data and cross-condition GI correlation. Related to Figure 7. **(A–E)** Violin plots of Pearson correlation distributions for sgRNA-level interaction profiles across the five GI/eGI maps. Gray violins: sgRNAs targeting different genes. Purple violins: sgRNAs targeting the same gene. Median values indicated beside each distribution. **(F)** Same analysis as (A–E), using the consensus matrix from Figure 7A. **(G)** Number of clusters formed from the consensus matrix as a function of Pearson distance. Red line indicates the threshold used (Pearson distance = 0.55). **(H)** Scatterplot of gene–gene GI profile correlations comparing either two reference maps or one reference and one environmental map. Red points: gene pairs from the same conserved cluster (Figure 7B). Dark red points highlight gene pairs in the mitochondrial homeostasis cluster for the glucose deprivation vs. second reference map comparison.
